# Designing Nanoparticles and Nanoalloys for Gas-Phase Catalysis with Controlled Surface Reactivity Using Colloidal Synthesis and Atomic Layer Deposition

**DOI:** 10.3390/molecules25163735

**Published:** 2020-08-15

**Authors:** Valentijn De Coster, Hilde Poelman, Jolien Dendooven, Christophe Detavernier, Vladimir V. Galvita

**Affiliations:** 1 Laboratory for Chemical Technology (LCT), Ghent University, Technologiepark 125, 9052 Ghent, Belgium; valentijn.decoster@ugent.be (V.D.C.); Hilde.Poelman@UGent.be (H.P.); 2 Department of Solid State Sciences, CoCooN, Ghent University, Krijgslaan 281/S1, 9000 Ghent, Belgium; Jolien.Dendooven@UGent.be (J.D.); Christophe.Detavernier@UGent.be (C.D.)

**Keywords:** heterogeneous catalysis, supported nanoparticles, controlled catalyst synthesis, area-selective atomic layer deposition

## Abstract

Supported nanoparticles are commonly applied in heterogeneous catalysis. The catalytic performance of these solid catalysts is, for a given support, dependent on the nanoparticle size, shape, and composition, thus necessitating synthesis techniques that allow for preparing these materials with fine control over those properties. Such control can be exploited to deconvolute their effects on the catalyst’s performance, which is the basis for knowledge-driven catalyst design. In this regard, bottom-up synthesis procedures based on colloidal chemistry or atomic layer deposition (ALD) have proven successful in achieving the desired level of control for a variety of fundamental studies. This review aims to give an account of recent progress made in the two aforementioned synthesis techniques for the application of controlled catalytic materials in gas-phase catalysis. For each technique, the focus goes to mono- and bimetallic materials, as well as to recent efforts in enhancing their performance by embedding colloidal templates in porous oxide phases or by the deposition of oxide overlayers via ALD. As a recent extension to the latter, the concept of area-selective ALD for advanced atomic-scale catalyst design is discussed.

## 1. Introduction

With an approximate contribution of 80–90% to all chemical processes [1], heterogeneous catalysis, wherein a gas- or liquid-phase reaction takes place over a solid catalyst, is indispensable in today’s society. Supported nanoparticles (NPs), which can be either metals or metal oxides, are particularly attractive in this discipline as they combine the high activity related to the catalytically active sites on the NP surface with the inherent thermal stability of support materials [2,3,4]. More so, interactions at the metal-support interface, called metal-support interactions (MSI), can significantly alter the catalytic behavior of the NPs, further proving the significance of these material combinations [5,6,7].

For a given metal and support, the catalytic activity, which is commonly expressed as turnover frequency (TOF), product selectivity and stability are dependent on the active sites exposed on the NP surface. In turn, these are a function of the NP size, shape, and composition [8,9,10,11,12,13]. To advance catalyst development, it is primordial to unambiguously establish correlations between these structural parameters and the catalyst’s performance. Such property-performance relationship studies help identify those catalyst properties that are responsible for enhanced catalyst performance, which, in turn, forms the basis of a knowledge-driven design of novel improved catalysts. However, such an approach requires model catalysts prepared by synthesis strategies that allow independent control over the structural parameters.

While single crystals have well-defined surface characteristics, these model materials are far from the more complex supported NPs that make up industrially relevant solid catalysts, thus making them unfit for investigating the many factors affecting catalytic behavior [14,15,16]. Moreover, single crystal studies are mostly performed under ultra-high vacuum (UHV) conditions [14]. This is in strong contrast with true reactive conditions which are closer to ambient pressures. These differences between model versus true materials and UHV versus elevated pressure testing conditions are respectively denoted as the “material gap” and “pressure gap” [16,17]. To cross these gaps, the synthesis of well-defined supported NPs is required. However, traditional wet chemical catalyst synthesis methods for the creation of such materials, such as wet impregnation (WI) [3,18], (co-)precipitation [3,19], ion exchange [18], or deposition-precipitation (DP) [20], mostly result in ill-defined property distributions, making them unsuitable for controlled catalyst synthesis.

To achieve the desired level of control, the application of colloidal NPs has proved an efficient strategy. Recent developments in wet chemical colloidal synthesis protocols have resulted in a high level of control over the size, shape, and composition of colloidal NPs [11,21,22,23]. As such, the use of metal colloids as a component of catalyst design, e.g., by the deposition of separately synthesized colloids onto a support, has gained increased attention for the controlled creation of catalysts with the appropriate level of complexity. Furthermore, recent developments have led to protocols for the creation of “embedded” NPs wherein the colloidally prepared NPs are enveloped by the support rather than supported onto it, which has opened a novel chapter for highly stable catalysts [4,24,25,26,27].

Atomic layer deposition (ALD), a self-limiting layer-by-layer growth method widely used for the fabrication of microelectronic devices, is another technique that has been successfully applied to achieve atomic-level control for catalyst synthesis [28,29,30,31,32,33]. Since the first reports of noble metal ALD in 2003 [34,35,36], this technique has gained interest for catalytic applications. Today, ALD-based catalyst synthesis has advanced to the creation of non-noble metal-containing bimetallics as well as the deposition onto porous supports, which are highly relevant in catalysis given their high surface area [29]. Moreover, deposition procedures of coatings over pre-synthesized NPs now offer additional routes to tailor the catalyst’s performance [28,32,37,38]. Due to its surface-controlled nature, ALD also has potential for selective deposition onto regions with a particular surface termination. By targeting specific surfaces of supported NPs, such “area-selective ALD” [33,37,38,39] shows great promise for the precise tailoring of the structural parameters, interfaces, and active sites.

This review covers recent progress in the application of catalysts with uniform properties prepared by colloidal synthesis and ALD in gas-phase catalysis. For both techniques, general principles of the synthesis procedures are first elaborated, followed by catalytic applications of mono- and bimetallic supported NPs prepared via these techniques. In view of the importance of improving catalyst stability, applications of catalysts with oxide-coated NPs are discussed. For ALD, the potential of area-selective ALD as a means for next-level atomic-scale catalyst design is highlighted. Finally, concluding remarks are given regarding the state-of-the-art of catalytic applications of these synthesis techniques, along with an outlook on their utilization and development in future studies.

## 2. Colloidal Synthesis in Gas-Phase Catalysis

### 2.1. Principles of Colloidal Synthesis for Supported Catalyst Preparation

By definition, a metal colloid, also called a “sol”, denotes a suspension of metal particles, typically in the range of 1–1000 nm, suspended in a liquid medium [40,41]. Herein, the particulates are covered by a protective layer that prevents them from coalescing into larger aggregates. The use of colloids is one of the most versatile methods in tuning catalyst properties, as it allows the creation of metallic, i.e., zerovalent, NPs independently of the support. Hence, this synthesis method allows for great tunability of the catalyst’s properties, provided the NPs are created in a controlled manner.

Methods for creating colloidal metals are classified in two categories: “top-down” or “bottom-up” [42]. Top-down refers to physical approaches that create NPs from the dispersion of bulk materials into their NP building blocks. Examples include, but are not limited to, laser ablation [43], chemical etching [44], and mechanical milling [45]. While such approaches usually produce relatively large quantities of NPs (~g), this advantage is countered by their broad particle size distributions, low synthesis reproducibility and the relative complexity of the instrumentation involved [21]. Bottom-up approaches, on the other hand, entail chemical routes whereby atoms or molecules are assembled into larger nanoscale (1–100 nm) structures. For the purpose of catalyst synthesis, the most common approaches are chemical reduction of metal precursors, i.e., where an external reagent is used to establish the reduction, and decomposition of organometallic precursors via thermal, photochemical, sonochemical, or radiolytic means [46], among which thermal decomposition is the most established [41]. While bottom-up methods yield smaller quantities of NPs (~mg) than top-down, they have the benefit of allowing better control over the NP morphology, a higher level of synthesis reproducibility as well as requiring less sophisticated equipment [21]. The aforementioned factors motivate the use of bottom-up approaches for colloidal synthesis of well-controlled supported NPs. Consequently, only these will be covered in this review.

**Figure 1:**
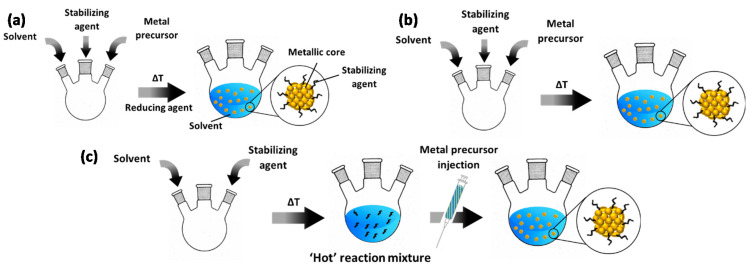
Common protocols for colloid synthesis: (**a**) chemical reduction; (**b**) thermal decomposition with precursor added in the initial step; (**c**) thermal decomposition via hot injection. The blue-green content of the syringe indicates that the metal precursor (green) is dissolved in the same solvent as the reaction vessel (blue). For ease of representation, the final step in the synthesis, i.e., cooling down of the reaction vessel, is not included.

Bottom-up colloidal synthesis relies on the controlled natural growth of suspended NPs. In a typical chemical reduction-based synthesis (Figure 1a), this entails the dissolution of precursors of the desired metal into a solvent in the presence of a protective agent. In the following step, the reduction process proceeds at elevated temperature to generate metallic NPs by introducing a reducing agent. In the case of protocols based on thermal decomposition, two common approaches are applied. The first, represented in Figure 1b, is near-identical to the chemical reduction approach described above; it only differs in the fact that no reducing agent is applied at elevated temperature and that this temperature is mostly higher than in the case of chemical reduction methods, e.g., ~200 °C versus ~100 °C. Another protocol relies on “hot injection” of the metal precursor (Figure 1c). Herein, solely the solvent and protective agent are first added to a reaction vessel. After heating this mixture, the precursor, typically dissolved in the same solvent as present in the reaction vessel, is injected into the ‘hot’ reaction mixture. After a certain dwell time at the final temperature, the synthesis is terminated by cooling off the system. 

From the above, it follows that bottom-up synthesis protocols comprise three key components: a metal precursor, a solvent, and a protective agent [13]. In the case of chemical reduction protocols, an external reducing agent is distinguished as an additional fourth component. It should be noted that these components are not necessarily physically separate; a certain chemical may act as more than one component. An example thereof are alcohols, which can act as solvent, protective agent and reducing agent [47,48]. 

The most common metal precursors are ionic salts, examples of which are nitrates, chlorides, sulfates, acetates, and acetylacetonates [13]. However, organometallic complexes are an equally viable option. Solvents provide the continuous liquid phase of the colloid and are either aqueous or organic. Protective agents envelop the particulates through interaction (e.g., adsorption or coordination), forming a protective shell with a twofold purpose [49]. First, it regulates the growth of the preformed NPs by the controlled diffusion of precursor from the surrounding liquid phase through this shell into the colloid’s core. Moreover, this protective layer prevents NP agglomeration through its stabilizing role, which is steric, electrostatic or electrosteric in nature [40,46]. Due to the the growth-controlling and stabilizing roles of protective agents, they are also called capping or stabilizing agents. A diverse range of such agents has been used in colloidal synthesis, common ones being linear (e.g., polyvinylpyrrolidone (PVP) [50]) or dendrimeric polymers (e.g., polyamidoamine (PAMAM) [51]), which adsorb onto the NP surface and impede coalescence through steric interaction with similarly protected NPs. Another frequently used option is ligand stabilization, referring to the use of molecules with functional groups (e.g., thiols [52], amines [53], organic acids [54], and phosphines [55]), wherein the heteroatom coordinates with the metallic atoms of the NP, thus providing steric stabilization. Microemulsions [56] or reverse micelles [57] through the use of surfactants are also widely applied. In view of chemical reduction, common reducing agents include [46]: alcohols (e.g., glycerol), gases (e.g., H_2_), hydrides (e.g., NaBH_4_), superhydrides (e.g., LiBEt_3_H), amine borane complexes (e.g., borane tert-butylamine complex (BTB)), and hydrazine.

The monodispersity, i.e., the uniformity in NP properties, inherent to bottom-up approaches arises from the separation of a short burst nucleation, wherein many nuclei are created at the same time, and the subsequent growth of these nuclei into NPs without additional nucleation events [13,58]. This can be achieved by a stepwise change in the synthesis environment, such as the addition of a reagent to the synthesis vessel. For chemical reduction methods, it is proposed that the injection of an external reducing agent into a mixture of precursor, solvent, and protective agent leads to the formation of many zerovalent nuclei in a short time [59,60]. The incorporation of remaining precursor from the surrounding liquid phase into these nuclei results into precursor reduction and the growth of these NPs. For thermal decomposition of organometallic salts, this separation of nucleation and growth is implemented by either quickly injecting the precursor into a hot (~200 °C) solution containing the capping agent or by imposing a controlled heating ramp to a solution that already contains the capping agent and precursor [61]. The decomposition of said precursors leads to an oversaturation of the solution, which is relieved by nucleation of metallic NPs. This is accompanied by the formation of decomposition products such as CO_2_ or H_2_O. As this initial nucleation event decreases the precursor concentration below the critical concentration for nucleation, remaining precursor material can only be further assimilated into the existing nuclei, thus preventing additional nucleation events. 

In either method, the protective agent in the reaction mixture ensures the controlled growth of the NPs after the short nucleation event, thus further assuring NP monodispersity. The interaction of the protective agent with the metallic core determines the strength of stabilization by the protective shell. Molecules that are larger (‘bulkier’) and bind more strongly with the metallic core provide greater steric hinderance, slowing down the rate of material addition and decreasing NP size. In combination with the protective agent, the solvent is indispensable in tailoring the colloid’s stability since a favorable interaction between these two components provides an extra barrier to counter NP coalescence.

The precursor material determines the underlying chemistry in the synthesis, such that changing the type of precursor may change the overall NP properties. However, the size, shape and composition of the NPs can be adapted by adjusting several experimental parameters [13,40,61]: the applied temperature; time; the type of protective agent, reducing agent, precursor, and solvent used; and the concentrations—or relative molar ratios—of the aforementioned components. Increasing the synthesis temperature promotes the kinetically driven incorporation of precursor material into the NPs as well as thermodynamically-driven particle growth by Ostwald ripening. Increasing the time spent at elevated temperatures leads to more precursor material being deposited into the NPs, and hence also results in larger NP formation. Upon reaching a set time or temperature, the synthesis vessel is cooled down by removing the heating source or by placing it in a cooling bath, stopping the growth. Increasing the molar ratios of protective agent/precursor favors the creation of more small nuclei, decreasing the NP size. The same effect can be attained through the use of stronger reducing agents. Moreover, protective agents can tailor the NP shape (e.g., spherical, cubic, tetrahedral) by influencing the growth direction through selectively adsorbing onto specific facets of a NP. NP composition is a property exclusive to bi- or multimetallic NPs. Tailoring the composition, i.e., the relative ratios of the elements making up the NP, can be achieved by varying the ratio of precursors in the synthesis or the order of addition to the synthesis vessel, e.g., simultaneously (co-reduction) or sequentially (successive reduction).

**Figure 2:**
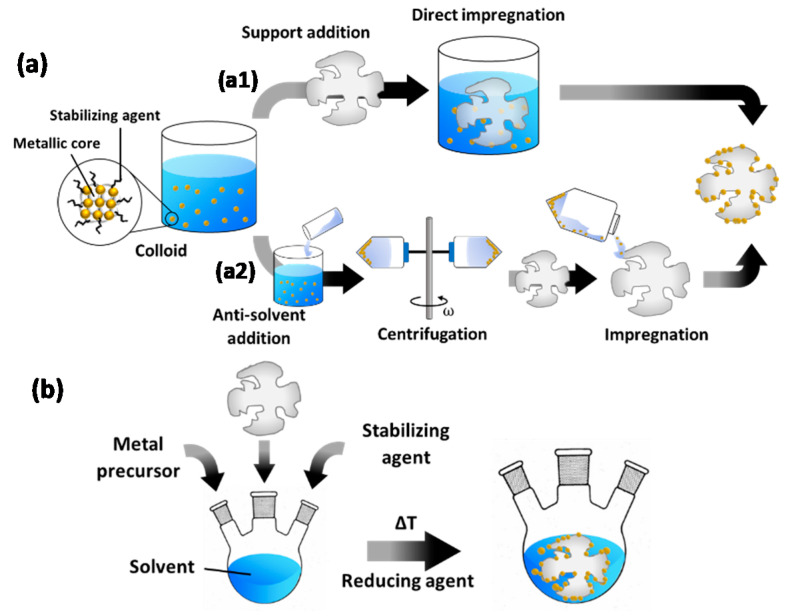
Common methods for supported catalyst preparation via colloidal synthesis. (**a**) Impregnation methods: (**a1**) direct impregnation by support addition to the prepared colloid; (**a2**) an anti-solvent is first added to the colloid, which stimulates NP flocculation, followed by centrifugation of the mixture (with angular velocity ω), and impregnation of the support with the centrifuged supernatant or precipitate phase. (**b**) In situ reduction method.

To create supported catalysts, colloids can be deposited onto the support by various methods [46,47]. The two most common ones are elaborated here and illustrated in Figure 2. In a first (Figure 2a), pre-synthesized colloids are impregnated onto the support. To this end, the as-prepared colloids can be used. However, since the NP concentration in these mixtures is very low, diffusion-driven migration of the NPs onto the support may take a long time. Adsorption can be stimulated by tuning the pH to promote electrostatic interaction between the NPs and the support [62]. Sonication can also stimulate NP immobilization on the support surface. This relies on the fact that shock waves, which are generated by the collapse of cavitation bubbles, push the NPs towards the support surface with high velocities and thus stimulate NP collision with the support [63,64]. Another option is an intermediary treatment, whereby the colloid is first destabilized through addition of an anti-solvent, resulting in NP flocculation, and subsequent centrifugation [65]. Contrary to the use of as-prepared colloids, this allows for “size-selective precipitation” [66] as it stimulates the precipitation of larger NPs, while smaller ones remain within the supernatant. The selected phase can then be dispersed in an anti-solvent followed by repeated centrifugation to fine-tune the particle size distribution. The final selection of NPs is dispersed in a solvent and impregnated onto the support.

The second method (Figure 2b), denoted as “in situ reduction”, involves the reduction of metal precursors in a mixture that already contains the support, which eliminates the need for a deposition step. In this case, the burst nucleation event is either heterogeneous in nature, i.e., occurring on the support, or forms metallic nuclei in solution, followed by their migration onto the support. 

Colloids can also be immobilized by grafting them onto the support [46]. This entails functionalizing the support such that the created groups either form chemical bonds with the protective shell or coordinate with the metallic core. While this yields strong immobilization, these approaches have the drawback that the organic-based chemistry requires complex modification steps and that the typically used sulfur-containing modification compounds can poison the catalyst [67,68].

The spatial distribution of the deposited NPs within the support depends on various factors. In the case of methods which rely on pre-synthesized NPs, the average NP size and support pore size are important parameters; the synthesized NPs must be smaller than the pores in order not to block diffusion-driven transport of the colloid into the support’s pores. If this is not the case, only the exterior surface of the support will be enriched in nanoparticulate material. Should the aforementioned requirements be met, it might still be necessary to wait a long time for the NPs to migrate into the pores as the diffusion of NPs is relatively slow [3,69]. This can be alleviated through sonication of the colloid-support mixture. Herein, the sonication-induced cavitational shock waves not only stimulate NP immobilization on the support surface, but also prevent NPs from blocking the pore openings [69]. In combination with the enhanced mass transport originating from these cavitation phenomena, this allows homogeneous dispersion of the NPs throughout the support channels-provided appropriate sonication times (~h) are applied [69]. In the case of in situ synthesis, the small size of precursor molecules (relative to the NPs formed from them), makes pore size not as stringent as in the case of methods relying on pre-formed NPs. Still, the distribution of the dissolved metal precursors within the support pores is a determining factor for the final spatial distribution of the NPs. Assuming the precursor is well-dissolved in the applied solvent, it follows that the extent of infiltration of the liquid into the pores is a critical factor in achieving homogeneous metal distributions. However, it has been proved that wetting liquids can infiltrate millimeters of a porous body in a matter of seconds or minutes [70]. Hence, attaining well-dispersed metal distributions through in-situ synthesis can be considered less problematic than in the case of pre-synthesized NPs. A detailed description on the factors affecting NP and metal precursor spatial distribution is beyond the scope of this work. For more information, reference is made to dedicated literature [3].

In a final preparation step, supports with deposited NPs are separated (e.g., by filtration), dried (e.g., in air, in vacuo or freeze-dried) to remove remaining solvent, and finally subjected to a thermal treatment. This treatment aims to remove residual stabilizing agent poisoning the active sites and to bring the catalyst into its active state, which can be either a zerovalent metal or a metal oxide or -sulfide [50,71,72,73,74,75]. However, care must be taken in choosing the conditions, particularly the temperature, of this ‘activation’ as it can induce redistribution of the NPs within the support, as well as phase segregation and sintering, which affect the NP morphology and, in turn, the catalytic performance [3,74,75].

### 2.2. Monometallic Supported Catalysts by Colloidal Synthesis

Significant progress has been made in the synthesis of monometallic colloids, such that it is now possible to create NPs of any element—provided an appropriate precursor exists—with tunable size and shape [4,21,58]. Specifically, in the field of heterogeneous catalysis, this has allowed for investigating the effect of the aforementioned properties on catalyst performance for a wide range of gas-phase reactions. 

#### 2.2.1. Oxidation Reactions

Low-temperature CO oxidation to CO_2_ is a catalytic reaction that is considered a “probe” or model reaction for other oxidation reactions and has essential applications in emission abatement [76]. In the near-50 years that this reaction has been studied, supported noble metals are among the most efficient reported catalysts [77]. For Pt catalysts, it has been established that interactions of this metal with the support can significantly alter the metal’s electronic properties and, consequently, the catalytic properties. To deconvolute the effects of MSI from NP size effects on the performance of the catalyst, the use of colloidal synthesis as a means to create monodisperse NP size distributions presents an opportunity. In recent work, Xi et al. [78] applied this strategy to study the support effect for Pt/CeO_2_ and Pt/SiC in CO oxidation. Materials were prepared by support impregnation with Pt NPs of 5.1 nm average size and subsequent calcination in air at 200, 400, 600, or 800 °C for 3 h. For higher calcination temperatures, as-prepared Pt/SiC displayed significant NP agglomeration, while this occurred to a much lesser extent for Pt/CeO_2_ samples. This was attributed to MSI through the formation of Pt-*O*-Ce bonds, as indicated by H_2_ temperature-programmed reduction (H_2_-TPR) and X-ray photoelectron spectroscopy (XPS). Similar bonds were not found for the SiC samples. In oxidative reaction studies, these interactions had a dual effect. For Pt/CeO_2_ calcined at 200 or 400 °C, the bonds were weaker, such that these provided active oxygen for CO oxidation without ‘eliminating’ Pt^0^, which is the active site for CO adsorption. For higher calcination temperatures, the Pt-*O*-Ce bond became stronger, resulting in the absence of Pt^0^, thereby deactivating the catalyst and yielding lower TOF values than Pt/CeO_2_ treated at lower temperatures. As no significant interactions between Pt and SiC were discerned, Pt/SiC calcined at 200 °C yielded lower activities compared to Pt/CeO_2_ that was treated similarly. However, after calcination up to 800 °C, Pt^0^ remained abundant in Pt/SiC, as opposed to Pt/CeO_2_, resulting in higher TOF values for the former catalyst. 

In the same context of oxidation reactions, the selective catalytic oxidation of alcohols to aldehydes, ketones and organic acids is an essential reaction in the creation of products with wide applications in the fine-chemicals sector [79]. For the purpose of selective benzyl alcohol oxidation to benzaldehyde, Kumar and co-workers [80] prepared 1 wt% Au/SBA-15 nanocatalysts through colloidal synthesis, as well as through three other synthesis methods, namely homogeneous deposition-precipitation (HDP), microemulsion, and WI, to investigate the effect of the preparation method on the NP dispersion and the catalytic performance. Specifically, for the colloidal method, supported Au NPs were created in situ via a polyol method, i.e., through the use of polyvalent alcohols (polyols). X-ray diffraction (XRD), H_2_-TPR and transmission electron microscopy (TEM) revealed that the synthesis method increased the NP size in the order HDP < ME < WI < colloidal synthesis, and that the NP dispersion increased in the reverse order. These parameters were correlated with the catalytic performance: HDP-prepared Au/SBA-15 yielded the highest benzyl alcohol conversions and benzaldehyde selectivities of all samples in the temperature range 280–360 °C. Even after regeneration, HDP catalysts still displayed the highest activity of all samples, though the activity was lower compared to the fresh catalyst. This was attributed to a decrease in the number of active sites of Au caused by NP agglomeration, which corroborated TEM images of the spent catalysts.

#### 2.2.2. Hydrogenation Reactions

A comparative study of synthesis methods was also performed by Saadatjou et al. [81] for Rh/γ-Al_2_O_3_ with respect to ammonia synthesis. Catalysts were prepared by incipient wetness impregnation (IWI) and an in-situ polyol reduction method. Notable is that the NP size of the IWI catalysts was larger (~12 nm) and more irregular than that of the polyol-synthesized variant (~7 nm). Subsequent reduction and activity measurements yielded the highest activities for the polyol samples. Accounting for the structure-dependency of NH_3_ synthesis on Ru, this was attributed to the smaller NP size and lower degree of contamination of the metal by aluminum from the support and precursor-related chloride. 

**Figure 3:**
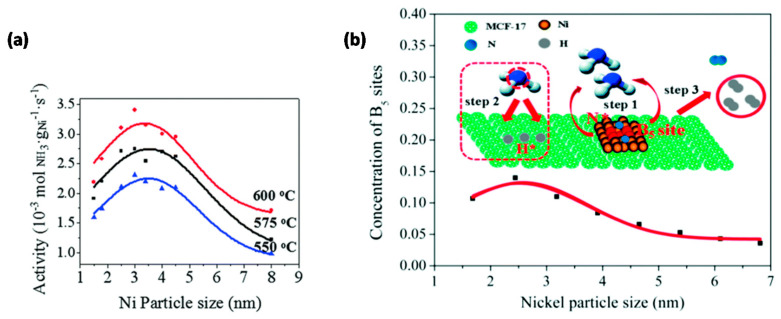
(**a**) NH_3_ decomposition activity of Ni/MCF-17, normalized to the Ni loading, for various reaction temperatures as a function of the average Ni NP size. Reaction conditions: P = 1 bar, pure NH_3_, GHSV = 6000 mL g_cat_^−1^ h^−1^. (**b**) The concentration of active B_5_ sites as a function of the average Ni NP size, with NH_3_ decomposition reaction mechanism on B_5_ sites of Ni catalysts. Step 1: NH_3_ chemisorption; step 2: consecutive dehydrogenation of NH_3_; step 3: associative desorption of H* and N* to form H_2_, respectively, N_2_. N* and H* denote adsorbed N and H atoms. Reproduced from Ref. [81] with permission from the Royal Society of Chemistry.

In recent years, the reverse reaction of NH_3_ synthesis, i.e., NH_3_ decomposition into N_2_ and H_2_, has become a topic of interest for CO_x_-free H_2_ fuel cells for clean energy [82,83,84]. Li and co-workers [85] confirmed the size-dependency of NH_3_ decomposition on Ni/MCF-17 through the use of uniform NPs with size distributions from 1.5 to 8 nm, tailored by adjustment of the colloid synthesis temperature. A volcano-type relationship was observed between the activity and the Ni NP size with a maximum at ~3 nm for reaction temperatures in the range 550–600 °C (Figure 3a). Theoretical calculations correlated this behavior with a maximum concentration of B_5_ sites, consisting of five atoms exposing a three-fold hollow hexagonally close-packed site and a bridge site close together, at ~2.5–3 nm (Figure 3b). Such B_5_ sites have been denoted in literature as the active sites in Ni-based NH_3_ decomposition catalysts [84,86,87]. 

Apart from the hydrogenation of N_2_ to NH_3_, Fischer-Tropsch synthesis (FTS), i.e., the conversion of a mixture of CO and H_2_ (syngas) into ‘long’ carbon-chain products, is one of the longest-studied reactions in chemical industry [88,89]. In view of replacing fossil resources with alternative, renewable feedstocks, this reaction has regained interest as it enables the conversion of syngas derived from these alternative resources into clean fuel [90,91]. However, challenges in the design of FTS catalysts remain, more specifically, in the creation of materials that are active, stable, and highly selective towards products with a narrow chain length distribution. As the structure-dependency of FTS is established, the level of control inherent to colloidal synthesis presents an opportunity for fundamental studies and, in turn, rational FTS catalyst design.

**Figure 4:**
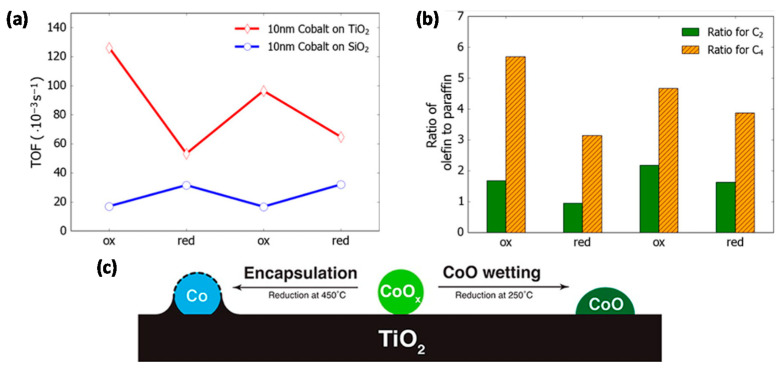
(**a**) TOF values for Co/TiO_2_ and Co/SiO_2_ FTS catalysts as a function of the oxidation state. (**b**) Olefin to paraffin ratios of C_2_ and C_4_ hydrocarbons for the Co/TiO_2_ FTS catalyst. (**c**) Schematic representation of the reversible TiO_2_ encapsulation of Co in Co/TiO_2_.“Ox” denotes oxidized Co, “red” reduced Co. Reduced Co was obtained via a H_2_ treatment. (H_2_: 10% (*V*/*V*), balance Ar) at 450 °C for 1 h, oxidized Co upon O_2_ treatment (O_2_: 20% (*V*/*V*), balance Ar) at 350 °C and maintained in a H_2_ atmosphere at 250 °C. Reaction conditions for (**a**) and (**b**): T = 250 °C, P = 5 atm, CO/H_2_ = 1/2, 24 h. Adapted with permission from Ref. [92]. Copyright 2014 American Chemical Society.

With regard to the application of colloidal synthesis for supported Co FTS catalysts, one of the first studies was performed by Melaet et al. [92], who prepared Co/TiO_2_ and Co/SiO_2_ with monodisperse Co NP sizes of ~10 nm. The activity towards FTS was twice as high for oxidized Co/TiO_2_ than for its reduced counterpart, while the contrary held true for Co/SiO_2_ (Figure 4a). In addition, this oxidation state influenced the product distribution, as more unsaturated products were formed for CoO/TiO_2_ (Figure 4b). In situ X-ray absorption spectroscopy (XAS), near edge X-ray absorption fine structure spectroscopy with total electron yield (NEXAFS-TEY), XRD and near-ambient pressure XPS (NAP-XPS) studies of Co/TiO_2_ indicated that this behavior originated from the reversible adsorbate-induced encapsulation of metallic Co by TiO_2-x_ species, illustrated in Figure 4c. Upon H_2_ treatment at 450 °C, Co is fully reduced, yet encapsulated by TiO_2_, limiting the fraction of exposed active Co sites. Upon O_2_ treatment, this encapsulation is reversed, and an accessible, catalytically active “TiO_2_-wetted CoO phase” is formed. Exposure of this CoO material to H_2_ at 250 °C did not induce TiO_2_ encapsulation nor CoO reduction.

The colloidal synthesis of Co/TiO_2_ FTS catalysts was also reported by Delgado and co-workers [93] to investigate the influence of NP size (1.7–7 nm) on catalytic performance. Smaller sizes resulted in higher activity and catalyst stability, which were correlated respectively with the higher reducibility of the smaller NPs and a promoting effect of residual boron, stemming from the NaBH_4_ reducing agent used in the synthesis [94,95,96]. In other work [73], the same group concluded that, for a given NP size, the reducibility is affected by the polymer stabilizing agent used in the synthesis. of the six water-soluble polymers investigated, poly(2-ethyl-2-oxazoline) resulted in the highest FTS activity.

**Figure 5:**
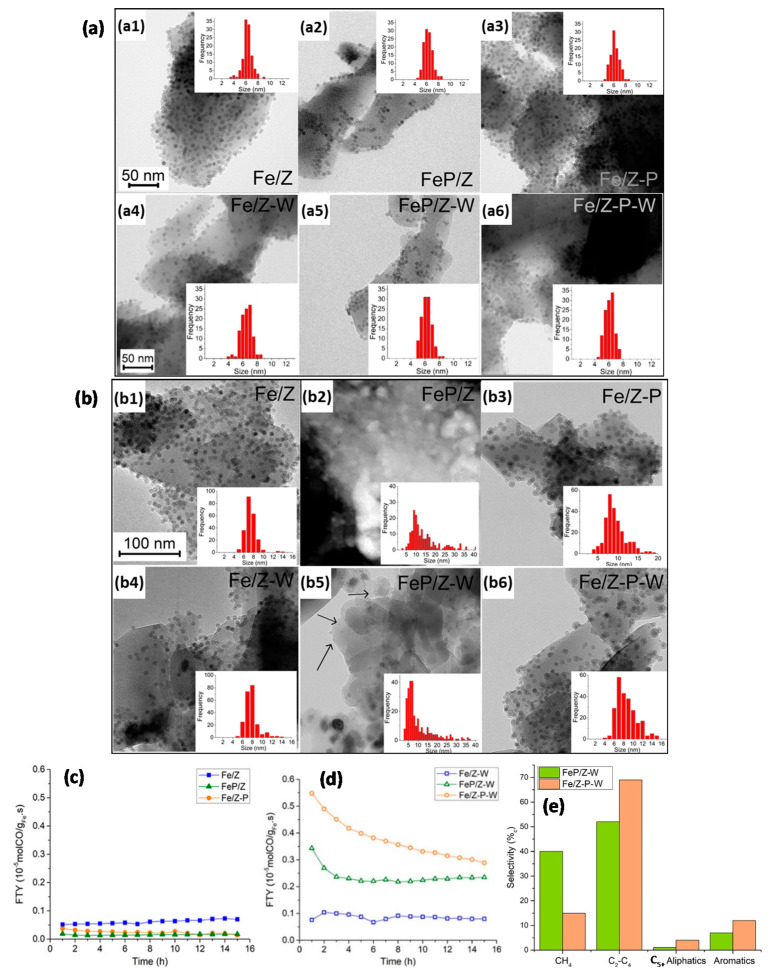
(**a**) TEM of as-prepared (**a1**) Fe/Z, (**a2**) FeP/Z, (**a3**) Fe/Z-P, (**a4**) Fe/Z-W, (**a5**) FeP/Z-W and (**a6**) Fe/Z-P-W. (**b**) Post-FTO TEM of (**b1**) Fe/Z, (**b2**) FeP/Z, (**b3**) Fe/Z-P, (**b4**) Fe/Z-W, (**b5**) FeP/Z-W and (**b6**) Fe/Z-P-W. Insets in (**a**) and (**b**) denote Fe NP size distributions. (**c**) Iron time yields (FTY, defined as moles of CO being converted per gram of iron per second) as a function of time-on-stream (TOS) for as-prepared, unwashed catalysts and (**d**) washed catalysts. Reaction conditions: T = 340 °C, P = 1 bar, H_2_/CO = 1/1 (*V*/*V*), CO conversion ~3–5%, GHSV = 5000 h^−1^. (**e**) Product selectivities of FeP/Z-W and Fe/Z-P-W in FTO conditions (T = 340 °C, P = 1 bar, H_2_/CO = 1 (*V*/*V*), GHSV: 4 200 h^−1^, TOS = 15 h). Adapted with permission from Ref. [97], https://pubs.acs.org/doi/abs/10.1021/acscatal.9b04380. Further permissions related to the material excerpted should be directed to the ACS.

In the development of FTS catalysts with high selectivity towards olefins, i.e., FT to olefins (FTO), and their further conversion into aromatics, Krans et al. [97] prepared Na_2_S-promoted Fe NPs anchored onto H-ZSM-5, denoted as “FeP/Z”. For comparison, unpromoted Fe NPs were also deposited onto H-ZSM-5 (“Fe/Z”). Subsequent Na_2_S promotion of this Fe/Z yielded “Fe/Z-P”. Overall, the aforementioned materials exhibited low FTO activities due to Na_2_S overpromotion (Figure 5c). To avoid detrimental overpromotion effects, the aforementioned materials were washed with an ammonia nitrate solution for ion-exchange, yielding “FeP/Z-W” and “Fe/Z-P-W”. This washing step was also applied to Fe/Z, resulting in “Fe/Z-W”. Now, FeP/Z-W and Fe/Z-P-W outperformed unpromoted Fe/Z-W (Figure 5d), producing aromatics and having selectivities over 50%_c_ towards C_2_–C_4_ fractions (Figure 5e). The methane selectivity was more than double for FeP/Z-W (40%_c_) than that for Fe/Z-P-W (15%_c_). Moreover, the promotion affected the particle growth during reaction. While notable Fe NP growth was observed for all promoted samples, whether or not washed, Fe NPs had grown most significantly for the FeP/Z catalysts (Figure 5a–b). This was ascribed to accelerated Ostwald ripening during FTO by the presence of promoters [98]. Consistently, inductively coupled plasma optic emission spectroscopy indicated the highest promoter-related Na and S content within FeP/Z, thus explaining the most notable NP growth in this sample. As expected from the lack of promoter elements in Fe/Z and Fe/Z-W, size distributions for these catalysts had changed insignificantly.

CO hydrogenation into ‘short’ carbon chain products is another option to valorize syngas originating from alternative resources. In a study on tuning the shape of Rh/ZrO_2_ catalysts for CO hydrogenation, van Hoof et al. [99] examined the effect of capping agent removability. Only for oleylamine (OAm)-stabilized NPs could thermal reductive treatments remove the larger part (89%) of the capping agent. When trimethyl(tetradecyl)ammonium bromide (TTAB) or PVP were used, residual capping agent covered 38–68% of the active Rh sites, as calculated from TEM and H_2_ chemisorption experiments. However, the resulting decrease in activity relative to the OAm-capped samples was limited, being 20–27%. The authors ascribed this to the fact that TTAB and PVP blocked mostly “non-critical”, i.e., less active, sites or that the conditions used in the experiments to determine the fraction of sites blocked are not representative for those under real CO chemisorption conditions. Noteworthy is that this ‘capping agent blocking effect’ greatly overshadowed any observable shape-induced changes in the activity. It is therefore important that the capping agent and catalyst thermal treatment are chosen such that the agent is removed for unbiased structure-reactivity studies.

Other than CO hydrogenation, the hydrogenation of CO_2_ has been the topic of many CO_2_ utilization studies. In the selective hydrogenation of CO_2_ to methanol (MeOH), Lam et al. [100] reported the synthesis of “Cu/Zr@SiO_2_” catalysts, i.e., wherein isolated Zr(IV) sites are first grafted onto SiO_2_ (“Zr@SiO_2_”) and Cu NPs are subsequently dispersed onto this material, to facilitate investigation of the interfacial interactions between Zr surface sites and supported Cu NPs. This catalyst showed an enhanced MeOH formation rate and selectivity compared to analogously prepared Cu/ZrO_2_ and Cu/SiO_2_. In-situ XAS and ex-situ solid-state nuclear magnetic resonance (NMR) spectroscopy indicated this improvement arose from the promotional effect of Zr(IV) sites at the support periphery activating CO_2_ and hydrogenating reaction intermediates.

### 2.3. Bimetallic Supported Catalysts by Colloidal Synthesis

The use of a secondary element in NP synthesis offers multiple advantages over monometallic NPs. In a first instance, interactions between the elements can increase the catalytic activity, yield, stability, and resistance towards poisonous species [101,102,103]. In the case of precious metals, the use of secondary, inexpensive elements can reduce the overall catalyst cost. Furthermore, bimetallic NPs introduce composition (i.e., relative abundance of each element over the whole NP) and architecture (e.g., random alloy, core-shell) as additional degrees of freedom [104], which provides more opportunities for catalyst improvement. Given the tunability inherent to colloidal approaches in controlling NP parameters, and thereby their performance, research on bimetallic catalysts prepared via such techniques has received much attention.

#### 2.3.1. Oxidation Reactions

**Figure 6:**
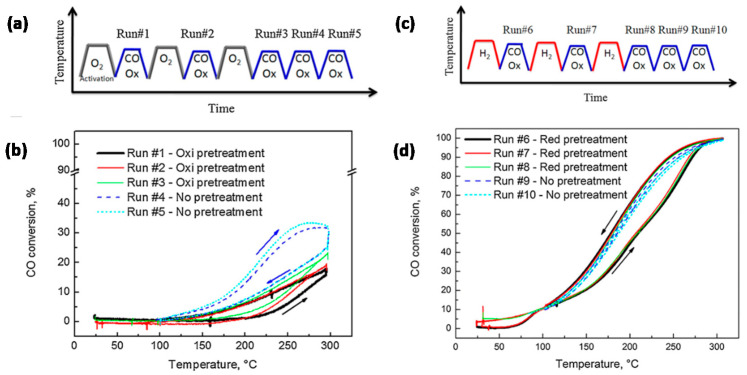
(**a**) Oxidation cycles coupled with CO oxidation tests applied to Au_0.75_Cu_0.25_/SiO_2_. (**b**) CO conversion results for the test protocol represented in (**a**). (**c**) Reduction cycles coupled with CO oxidation tests applied to Au_0.75_Cu_0.25_/SiO_2_. (**d**) CO conversion results for the test protocol represented in (**c**). Reaction conditions for (**b**) and (**d**): 1% CO (*V*/*V*), 6% O_2_ (*V*/*V*), He balance, GHSV = 3,000,000 Ncc/min (g of (Au + Cu)). Reprinted with permission from Ref. [106]. Copyright 2017 Elsevier.

In view of improving Au catalysts for CO oxidation by the addition of Cu, Destra et al. [105] studied AuCu catalysts (~10.2 ± 1.0 nm, in a fresh sample) on γ-Al_2_O_3_ and SiO_2_ supports, prepared via a colloidal co-reduction method. The catalysts’ performance was substantially influenced by oxidation (O_2_) and reduction (H_2_) cycles prior to reaction [106,107]. After each O_2_ cycle, the CO conversion in the subsequent activity test increased, up to 23% after three cycles (Figure 6a–b), and attaining 34% CO conversion after two subsequent reaction cycles without O_2_ pre-treatment (run #5 in Figure 6a–b). In contrast, after a single H_2_ cycle, the CO conversion already approached a maximum of 100% (Figure 6c,d). The significant enhancement after reduction by H_2_ was attributed to a more thorough purification of precursor-related poisonous Cl species under H_2_ than under O_2_. However, as reduction by H_2_ induced Au-Cu alloying and oxidation by O_2_ resulted in dealloying through the formation of separate CuO_x_ phases, it was not excluded that the different performances after O_2_ and H_2_ cycles resulted from the formation of structures with quite different reactivities [108]. Notable herein is that, after the first oxidation and reduction cycle, no notable changes occurred in the material’s particle sizes relative to the fresh sample. The corresponding NP size distributions amounted to 10.2 ± 1.2 nm (fresh), 9.9 ± 1.2 nm (oxidized) and 10.1 ± 1.0 nm (reduced).

Zaytsev et al. [109] prepared AuPd/γ-Al_2_O_3_ nanoalloys using various stabilizing agents (PVP, polyvinyl alcohol (PVA), Triton X-100 and AF-6 and AF-12 neonols) in the colloid synthesis. The CO oxidation performance of these catalysts was influenced most by the thermal treatment prior to reaction. For a given reaction temperature, calcination in dry air resulted in higher CO conversions than calcination in a H_2_ atmosphere or mere drying in air. This was ascribed to a more complete removal of the stabilizing agent, as corroborated by thermogravimetric analysis (TGA). However, calcination resulted in particle growth to 15–20 nm sizes, in contrast to the 3–6 nm nanocrystallite sizes of the catalyst prepared by drying. In addition, Triton X-100-stabilized NPs resulted in slightly higher activities, which was related to the stronger interaction of this surfactant with the support, thus enhancing NP immobilization and dispersion during reaction. 

Nagy et al. [110] studied Ag as the secondary metal phase for SiO_2_-supported Au catalysts for their application in benzyl alcohol oxidation and CO oxidation. They assessed the influence of NP composition and catalyst pre-treatment, i.e., calcination and successive reduction, on the catalytic performance. For benzyl alcohol and CO oxidation, the activity as a function of the molar Ag/Au ratio reached a maximum for Ag/Au = 23/77 in both the calcined and reduced samples, thus indicating a synergistic effect compared to monometallic Ag or Au. Furthermore, the oxidation state of the metal had a significant influence on the activity. In benzyl alcohol oxidation, a successive thermal treatment resulted in an activity increase, while this had a more complex effect in the case of CO oxidation. These changes were attributed to the fact that the treatments resulted in the creation of different types and quantities of active sites. The exact underlying nature of these sites is to-date not fully clear, however.

In the class of total oxidation reactions, the catalytic combustion of alkanes forms an indispensable element in the context of pollution abatement and energy efficiency improvement. Due to its high energy density and potency as a greenhouse gas, methane is one of the designated reagents for combustion [111]. PtPd bimetallic catalysts are among the most important methane combustion catalysts. The combined use of Pt and Pd can lead to PtPd alloy formation, for which both positive [112,113] and negative [114,115] effects on combustion activity have been reported. Qu et al. [116] used the level of control granted by colloidal methods to devise a strategy to synthesize a PtPd/Mg-Al_2_O_3_ catalyst without PtPd alloy formation. When compared to a catalyst with the same metal loading prepared by WI, the colloidally prepared material already exhibited ~90% methane conversions at temperatures as low as 400 °C, while conversion values below 10% were obtained for the WI sample at the same temperature. The authors attributed this excellent performance to the formation of a Pt-PdO structure that enhances the redox properties of the catalyst and removes poisonous OH groups during combustion. 

#### 2.3.2. Hydrogenation Reactions 

**Figure 7:**
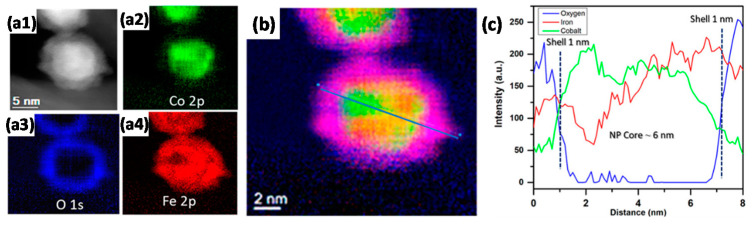
Janus-like CoFe alloy formation in CoFe/CNT FTS catalyst after reduction by H_2_. (**a1**) HAADF-STEM of CoFe/CNT catalyst; (**a2**)–(**a4**) corresponding EDX elemental mappings of (**a2**) Co, (**a3**) O and (**a4**) Fe. (**b**) Composite red-green-blue elemental mapping of Fe (red), Co (green) and O (blue), with EDX line scan pattern drawn on top. (**c**) EDX line scan profile corresponding to the pattern drawn in (**b**). Adapted with permission from Ref. [118], https://pubs.acs.org/doi/abs/10.1021/acscatal.8b04334. Further permissions related to the material excerpted should be directed to the ACS.

The addition of promoting elements is a known strategy for tuning the performance of FTS catalysts [117]. In an approach to study Co-Fe interactions and their effect on FTS performance, Ismail et al. [118] prepared monodisperse (5–7 nm) carbon nanotube (CNT)-supported CoFe catalysts via a nonhydrolytic colloidal method. The reduced catalyst displayed partial Janus-like alloy formation (Figure 7) and had higher activities and selectivities compared to monometallic counterparts. Moreover, through XRD and scanning TEM energy dispersive X-ray (STEM-EDX) analysis, the active phase in the bimetallic system for FTS was proposed to be a function of the employed reaction conditions, CoFe alloy and Fe- and Co-carbides being the identified phases in low- and high-temperature FTS, respectively. 

Dad et al. [119] studied the effect of Mn-promotion on Co via colloidally prepared CoMn supported on Stöber (SiO_2_) spheres. Apart from higher olefin selectivity, the catalyst proved more stable in FTS than unpromoted Co/SiO_2_. Not only the type of promoter but the NP architecture [120,121,122] can be used to tune the catalytic performance. Haghtalab et al. [123] applied a colloidal synthesis approach to prepare Co@Ru/γ-Al_2_O_3_ (supported core-shell NPs) catalysts with controlled Ru shell thickness. The electronic interaction between Co and Ru in the core-shell configuration resulted in higher CO conversion and selectivities towards desirable C_5+_ products than for monometallic Co and Ru catalysts. More so, a thicker Ru shell led to a higher increase in activity, illustrating the power of performance tunability achievable via colloidal techniques. 

In the application of generating clean fuels [124,125] and chemical products [126] from biomass-derived resources, the direct production of dimethyl ether (DME) from syngas is an attractive option. Generally, such syngas-to-dimethyl (STD) catalysts consist of Cu/ZnO, i.e., the active phases in a standard methanol synthesis catalyst [127], and a dehydration component, e.g., γ-Al_2_O_3_ [128] or H-ZSM-5 [129]. To further the understanding of these catalysts through fundamental studies of well-defined materials, Gentzen and co-workers [130] reported a method starting from bimetallic CuZn colloidal NPs for the preparation of Cu/ZnO/γ-Al_2_O_3_ STD catalysts, which proved highly reproducible and resulted in uniform NP properties. Tunability of both the methanol formation and dehydration functionalities via this method, allowed achieving CO conversions up to 24% and DME selectivities up to 68%. The same synthesis protocol was used to systematically investigate the effect of the acidic properties of the dehydration component (γ-Al_2_O_3_, H-ZSM-5 or HY) on the performance in one-step conversion of syngas to DME and hydrocarbons [131]. For given reaction conditions, the CO conversion, selectivities towards DME and C_1_–C_4_ products, and the DME formation rate could be tuned by varying the ratio of Cu to acidic sites as well as the micropore area. 

Based on the reported efficiency of PdZn phases for MeOH synthesis [132,133], Gentzen et al. [134] developed a colloidal protocol for the synthesis of Pd/ZnO-γ-Al_2_O_3_ as an alternative to the aforementioned CuZn-based STD catalyst. Most notably, STD activities, DME selectivity and catalyst stability were enhanced relative to conventional Cu/ZnO/γ-Al_2_O_3_. Following density functional theory (DFT) calculations and in situ and operando XAS, this was attributed to the presence of a stable intermetallic PdZn phase that is formed during catalyst activation and is the designated MeOH active component as part of the bifunctional STD catalyst.

Higher (C_2+_) alcohols are an equally viable CO hydrogenation product for a wide variety of day-to-day applications, e.g., fuels, cosmetics, polymers [135]. Bimetallic CuFe catalysts are a promising material for catalytic syngas conversion into such higher alcohols [136,137,138,139]. He et al. [140] created well-defined CuFe/CNT materials to study the structural factors that impact their performance in the formation of C_2+_ alcohols from syngas. They utilized pre-formed colloids prepared via stepwise- and co-reduction approaches and compared these with catalysts prepared through conventional WI. The catalyst prepared via co-reduction showed the highest C_2+_ alcohol selectivity (~21%). As the distance between Fe and Cu domains proved to be the smallest for this sample, as determined by high-angle annular dark-field scanning TEM (HAADF-STEM) imaging, it was proposed that the C_2+_ alcohol selectivity correlated with the proximity between the active Cu and Fe_2_C species. Quantum-chemical simulations corroborated this, as the energy barrier of the rate-determining step, determined as the C-C coupling to form CH_3_CHO (CH_3_ + HCO → CH_3_CHO), was higher when the contact between Fe and Cu species was less intimate.

**Figure 8:**
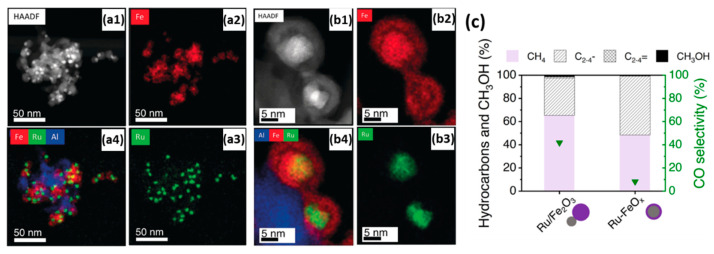
(**a1**) HAADF-STEM of Al_2_O_3_-supported Ru/Fe_2_O_3_ heterodimers after calcination; (**a2**)–(**a4**) corresponding EDX elemental mappings of (**a2**) Fe, (**a3**) Ru and (**a4**) composite Fe, Ru and Al mapping. (**b1**) HAADF-STEM of Al_2_O_3_-supported heterodimers after reduction by H_2_ at 300 °C and subsequent CO_2_ hydrogenation; (**b2**)–(**b4**) corresponding EDX elemental mappings of (**b2**) Fe, (**b3**) Ru and (**b4**) composite Fe, Ru and Al mapping. (**c**) Hydrocarbons and MeOH distributions and CO selectivities in CO_2_ hydrogenation for heterodimer Ru/Fe_2_O_3_/Al_2_O_3_ and core-shell “Ru-FeO_x_/Al_2_O_3_” catalysts reduced at 300 °C. Reaction conditions: 25% CO_2_ (*V*/*V*), balance H_2_, T = 300 °C, P = 6 bar, m_cat_ = 30 mg, CO conversion = 18% (Ru/Fe_2_O_3_), 19% (Ru-FeO_x_). Reproduced with permission from Ref. [141]. Copyright 2019 John Wiley and Sons.

For the purpose of investigating the promoting effect of Ru on Fe with respect to the selective hydrogenation of CO_2_ to hydrocarbons, Aitbekova et al. [141] synthesized well-defined Al_2_O_3_-supported “Ru/FeO_x_ heterodimers” (Figure 8a) via the successive growth of iron oxide NPs (~13.1 nm) on pre-synthesized Ru NPs (~4.8 nm). Through in situ XAS studies, the authors proposed that the synergistic effect between Ru and Fe is implemented via hydrogen spillover from Ru to neighboring Fe. Furthermore, after a reductive activation pre-treatment, the heterodimers transformed into “Ru-Fe” core-shell structures. Post-reaction TEM, represented in Figure 8b, revealed this structure persists during the reaction. However, the formation of this relatively thick (~4.3 nm) Fe shell introduced a catalytic behavior similar to that of a monometallic Fe catalyst, thus virtually eliminating the effect of Ru. These considerations led to the design of an Al_2_O_3_-supported “Ru-FeO_x_” core-shell catalyst with a thinner shell (~1.2 nm) through an adapted stepwise reduction protocol. Yields for this catalyst were four times higher than for the heterodimer Ru-Fe variant (Figure 8c). Post-reaction TEM revealed no morphology changes in the catalyst with a thin Ru shell. 

Within the selective hydrogenation reactions, the partial hydrogenation of alkynes via heterogeneous AuNi catalysts provides a cost-effective alternative to Pd-based materials [142]. Recently, Bruno and co-workers [143] reported a novel colloidal synthesis method for the preparation of Al_2_O_3_-supported AuNi NPs for application in 1-octyne partial hydrogenation. While bimetallic materials had activities and selectivities in between those of monometallic Ni and Au, their stability was significantly better than that of the constituent materials. The activity of Au and Ni catalysts halved within 20 h, whereas that of AuNi improved and remained stable for a week. Additionally, the tendency towards overhydrogenation was lower in the bimetallic catalysts, which was attributed to the presence of Au at the NP surface, suppressing H_2_ adsorption on Ni.

#### 2.3.3. Dehydrogenation Reactions 

The dehydrogenation of hydrocarbons to olefins is industrially exploited world-wide for the production of key building blocks for fuels, polymers, and fine chemicals [144]. With the goal of propane dehydrogenation (PDH), Sn-promoted Pt catalysts are particularly effective [145,146,147,148,149,150,151]. To investigate the extent of Sn promotion on Pt-based PDH catalysts, Kaylor and Davis [152] evaluated the influence of catalyst preparation method (colloidal versus IWI), Sn content and support (Al_2_O_3_ versus SiO_2_). As expected, colloidally prepared samples showed more monodisperse NP size distributions. The type of support affected the PDH performance similarly for both colloidally and IWI-prepared catalysts. Lower Sn loading resulted in less effective promoting effects in the case of Al_2_O_3_ than SiO_2_. When subjected to oxidative regeneration to remove coke formed during reaction, PtSn/Al_2_O_3_ recovered its activity while PtSn/SiO_2_ deactivated irreversibly due to significant PtSn dealloying by segregation of SnO_x_ to the catalyst surface. These observations were ascribed to stronger MSI for Al_2_O_3_ than SiO_2_.

#### 2.3.4. Reforming Reactions 

Dry reforming of methane (DRM) involves the simultaneous conversion of the greenhouse gases methane and CO_2_ into syngas, which can serve as building block for a wide variety of reactions. Due to its environmental and industrial relevance, research effort has focused on the development of active, stable, and cost-effective DRM catalysts. In that regard, supported NiFe catalysts have proved effective in meeting these demands [153,154,155,156,157,158,159,160]. In an effort to improve the DRM activity by downsizing the NPs, Margossian et al. [161] applied a colloidal strategy in the preparation of uniform NiFe/Mg(Al)O catalysts with selected Ni- and Fe loadings. The initial activity of Ni/Mg(Al)O prepared by this route was 10 times higher than for a WI-prepared sample, but lost 89% of activity after 30 h time-on-stream (TOS). In contrast, monometallic Fe was inactive. While TOF values of an optimized Ni_0.75_Fe_0.25_ were slightly lower than for the monometallic Ni catalyst, it deactivated much more slowly, losing 31% of activity after 30 h TOS. For all bimetallic catalysts, higher reduction temperatures resulted in lower activities due to inactive Fe enriching the catalyst surface. At lower temperatures, NiFe alloy formation was more profound. Under DRM conditions, the constituent Ni^0^ is the active site for DRM, while neighboring Fe forms FeO, which allows for the decoking of Ni [153,158,159]. 

### 2.4. Gas-Phase Catalysis by Embedded NPs 

While the use of colloids can indeed lead to high control over catalytic properties, deposited NPs obtained via this method typically suffer from structural instability upon exposure to thermal activation treatments and the reactive environment. For instance, metal sintering or dealloying can occur, compromising the structural integrity of as-synthesized colloids with correlated effects on the catalytic performance. In that regard, “embedding” the colloidal NPs within a support proves an interesting solution. By encasing the particles, their mobility is restricted, such that sintering-induced deactivation is mitigated [162]. The support’s porosity, created by thermal and/or chemical treatments [163,164,165], ensures the mass transfer of reagents and products. More so, tailoring the porosity of this supporting phase allows adapting the accessibility and rate at which certain molecules are transported, thus affecting the product selectivity and reaction rate [25].

**Figure 9:**
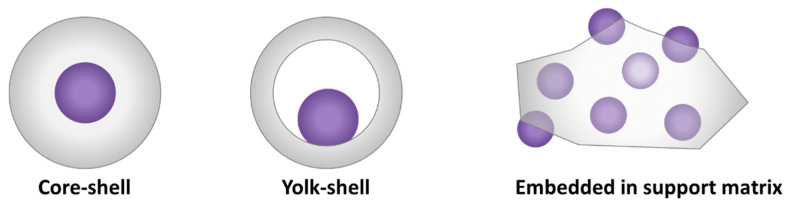
Common “embedded” NP catalyst architectures. Purple denotes the active phase, grey the support phase.

Different embedment architectures can be distinguished based on the spatial distribution of the active phase and the support. Common ones are represented in Figure 9. In core-shell architectures [24,25], the core consists of the active phase which is completely encapsulated by a porous oxide shell. Yolk-shell is another established option [24,166]. Analogous to a bird’s egg wherein an egg yolk is surrounded by albumen and a hard shell, each individual NP in this architecture is successively surrounded by a cavity and a porous shell. As the NP core is enveloped by a relatively homogeneous environment, this makes the NP’s active sites more accessible for reaction than in core-shell architectures. Furthermore, the use of a core with multiple shells is possible, as are many other variations [167]—a full enumeration of which is beyond the scope of this review. Apart from the deposition of porous layers onto each NP, colloidal NPs can also be embedded—partially or completely—within a support matrix [21]. The latter is thus in contrast with yolk/core-shell structures, as this does not lead to the individual encapsulation of NPs with a separate support layer, but rather to multiple NPs encapsulated in a common support phase. For a detailed description of the synthesis of these different architectures, the reader is referred to dedicated literature [21,24,25,27,167,168].

The wide array of embedment techniques for colloidal NPs does not only offer a means of stabilizing NPs, but adds additional tunability to the design of solid catalysts by adjustment of the support’s architecture and porosity. In this section, recent gas-phase catalysis applications of support-embedded catalysts prepared via a colloidal template are discussed. 

#### 2.4.1. Oxidation Reactions

**Figure 10:**
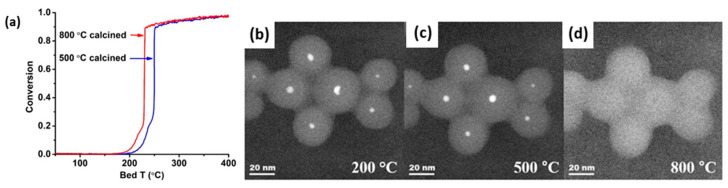
(**a**) CO oxidation light-off curves for Pd@SiO_2_ after aging in air at 500 °C (blue) and at 800 °C (red). Reaction conditions: 2 °C/min, 1% (*V*/*V*) CO, 1.5% (*V*/*V*) O_2_, balance N_2_, total flow rate = 200 mL min^−1^, m_cat_ = 60 mg. (**b**)–(**d**) In situ (S)TEM of Pd@SiO_2_ after aging in 150 Torr O_2_ at (**b**) 200 °C for 1 h, (**c**) 500 °C for 30 min and (**d**) 800 °C for 30 min. Reprinted with permission from Ref. [169]. Copyright 2018 Elsevier.

As stated before, CO oxidation is a probe reaction used to investigate the effect of composition and structure of the active phase under reaction conditions. In the case of embedded nanomaterials, it is therefore ideal to investigate the degree of structural stability induced by the encapsulation phase. Due to the structure-insensitivity of CO oxidation over Pd catalysts, Seo et al. [169] specifically opted for this reaction to evaluate the effect of aging Pd@SiO_2_ core-shell materials in air. Most notably, a better oxidation performance was observed when the material was aged at 800 °C instead of at 500 °C (Figure 10a). This originated from the partial redispersion of the ~4 nm Pd cores into smaller ~2 nm NPs within the SiO_2_ shells, which only occurred at temperatures above 800 °C, as revealed by in situ TEM imaging (Figure 10b–d). At 500 °C, the Pd mobility was too low to allow for this redispersion, such that the NP retained its original morphology. In contrast, a supported Pd/SiO_2_ catalyst underwent notable sintering at these temperatures. 

**Figure 11:**
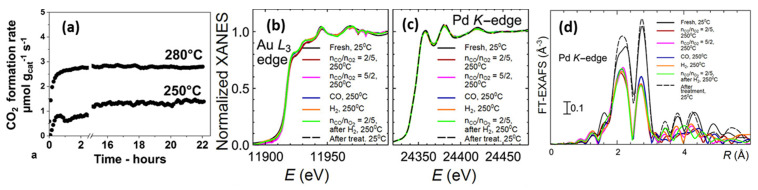
(**a**) CO oxidation stability test results of Pd_0.09_Au_0.91_ RCT-SiO_2_. Reaction conditions: 5% (*V*/*V*) CO, 10% (*V*/*V*) O_2_, He balance; GHSV = 2000 h^−1^, total flow rate = 25 mL min^−1^ ; m_cat_ = 40 mg. (**b**) In situ XANES spectra for the Au L_3_ edge and (**c**) the Pd K edge for Pd_0.09_Au_0.91_ RCT-SiO_2_ under reaction conditions. (**d**) Corresponding Fourier-transformed k^2^-weighted EXAFS spectra for the Pd K edge. Adapted with permission from Ref. [172]. Copyright 2019 American Chemical Society.

For the purpose of embedding NPs within a support material, catalyst synthesis via a raspberry colloid-template (RCT) is a promising method [170,171]. Such RCT materials are prepared by binding metallic NPs onto the surface of colloidal polymeric particles, which creates colloids with a “raspberry” morphology. Following the spontaneous assembly of these raspberry colloids, the interstitial spaces of this assembly are filled with metal oxide matrix precursor material. By subsequently subjecting this system to a heat treatment, the polymeric template is removed, leaving only a porous metal oxide with continuous, interconnected pores, decorated with metal NPs. Luneau and co-workers [172] studied PdAu NPs with dilute, i.e., low, Pd concentration (maximum 0.09 at%), partially embedded in raspberry colloid-templated-SiO_2_ (RCT-SiO_2_). They demonstrated the material’s stability under CO oxidation (Figure 11a). This latter aspect was corroborated by in situ XAS measurements as no notable changes in either Pd K or Au L_3_ edge features were observed under reactive conditions, indicating no net change occurred to the initial PdAu alloy state (Figure 11b–d).

Kim et al. [173] evaluated Pt@SiO_2_ core-shell materials in both CO and CH_4_ oxidation as a means to investigate its stability in low (<300 °C) and high (>500 °C) temperature regimes, respectively. While Pt@SiO_2_ achieved lower CO oxidation activity compared to supported Pt/SiO_2_, which was attributed to a lower number of exposed sites in the embedded material, similar activation energies were obtained for both materials, indicating an identical oxidation mechanism. Under CH_4_ oxidation conditions, Pt/SiO_2_ agglomerated, while Pt@SiO_2_ largely preserved its morphology, resulting in higher activity and stability. Still, deactivation occurred for Pt@SiO_2_, albeit to a lesser extent than in Pt/SiO_2_. This was hypothesized to be caused by temperature-induced shell degradation and Pt NP agglomeration. 

Given the high operating temperatures of catalytic combustion reactions, embedding NPs within oxide materials is an attractive way to enhance catalyst stability for these reactions. Habibi et al. [174] tested SiO_2_-encapsulated colloidal PtPd alloy NPs in wet (5 mol% H_2_O vapor) and dry catalytic CH_4_ lean combustion. The catalyst performance remained stable after 70 h under combustion conditions. Additionally, the catalysts were tested for stability after hydrothermal aging, which comprised a 50 h thermal treatment using the wet combustion feed. Hydrothermally aged SiO_2_-encapsulated structures achieved two- and ten-times higher conversions with respect to similarly treated PdPt/Al_2_O_3_ and PdPt/Al_2_O_3_ prepared via IWI. The enhanced performance was correlated with the higher dispersion of PtPd NPs within the SiO_2_ shell after aging. For the purpose of enhancing the thermal stability of Pt toluene combustion catalysts without inducing severe mass transport limitations, Pei and co-workers [175] developed an in situ synthesis method to partially embed Pt NPs into three-dimensionally ordered macroporous (3DOM) Mn_2_O_3_, whereby the Mn_2_O_3_ structure consists of a three-dimensional structure with ordered spherical macropores (>50 nm) which is prepared by a colloidal template method [176]. 2.3 wt% Pt/Mn_2_O_3_-3DOM exhibited the best catalytic performance. Due to the confining nature of this embedment, the stability, as tested for 60 h TOS, proved to be better than that of a similar material prepared via colloid adsorption. Consistent with these results, the average NP size of the embedded Pt catalyst changed insignificantly from 4.3 nm to 4.7 nm. 

Recently, Shirman et al. [171] made progress in catalyst design for selective alcohol oxidation by applying a partial embedment of AgAu NPs in RCT-SiO_2_ and assessing their performance in the oxidative coupling of MeOH and EtOH to produce esters. The robustness of these catalysts was proved by their stable catalytic performance. In accordance, TEM imaging confirmed the absence of NP sintering and agglomeration both after calcination and repeated activity tests with 40 h TOS. 

#### 2.4.2. Hydrogenation Reactions

While SiO_2_ encapsulation is an option to prevent the detrimental effects of NP agglomeration and sintering in Fe FTS catalysts, SiO_2_ suffers from low hydrothermal stability and can have adverse effects on the catalytic properties upon interaction with Fe [177,178]. In that regard, modification of SiO_2_ through the addition of promoters presents an opportunity. To weaken the Fe-SiO_2_ interactions, Ni et al. [179] applied graphitic carbon-promoted SiO_2_ (SiO_2_-GC). Core-shell Fe@SiO_2_-GC catalysts with optimized composition could achieve higher CO conversions and C_2_–C_4_ selectivities than an Fe@SiO_2_ core-shell catalyst after 100 h TOS. Conversion, stability, and selectivity improvements were attributed to the GC-promoted SiO_2_ shell. Due to the enhanced hydrothermal robustness by GC-promotion, the shell integrity was conserved under FTO conditions, such that Fe confinement and high Fe dispersion were maintained even over longer TOS. Moreover, the narrow pore size distribution of the SiO_2_-GC shell exerted a spatial-restricting effect that inhibited the formation of longer carbon chains and favored the formation of shorter (C_2_–C_4_) chain lengths. 

In the selective hydrogenation of CO into higher alcohols via CuFe catalysts, embedding NPs in porous metal oxides is ideal to maintain intimate contact between Cu and Fe species during reaction. A facile method for the encapsulation of pre-synthesized CuFe NPs in SiO_2_ shells was developed by Huang et al. [180]. The prepared structures were spherical in nature (average diameter 80–90 nm) and consisted of multiple CuFe cores in a shared SiO_2_ shell. In CO hydrogenation activity studies, CuFe@SiO_2_ materials outperformed unsupported CuFe NPs in terms of alcohol selectivity and CO conversion after 252 h TOS. Comparative pre-use and post-mortem TEM studies of the embedded material revealed the formation of dual functional Cu–χ-Fe_5_C_2_ during reaction, a phase which was maintained, i.e., without sintering or Cu-Fe phase separation, due to the restricting nature of the shell, thus explaining its enhanced performance. 

Ilsemann et al. [181] reported Co@SiO_2_ and Co@silicalite-1 catalysts for the methanation of CO, CO_2_ and CO/CO_2_ mixtures. Compared to Co/SiO_2_ prepared by WI, the colloidally-prepared embedded materials exhibited increased CH_4_ formation rate and selectivity in the order Co/SiO_2_ < Co@silicalite-1 < Co@SiO_2_. These observations were linked to the confinement by the silica phase, which is most pronounced in Co@SiO_2_. It was proposed that this effect stimulates the adsorption and hydrogenation of CO intermediates in the reactive environment near the active Co phase. Carbon-related deactivation occurred for all catalysts under methanation conditions. No sintering was observed for the core-shell catalysts. This is in contrast with Co/SiO_2_, whereby sintering induced an increase of the average NP size, as pre-and post-reaction NP sizes amounted to 39 nm and 44 nm, respectively. Hence, the absence of sintering in Co@SiO_2_ justifies the use of encapsulation for stability enhancement. 

**Figure 12:**
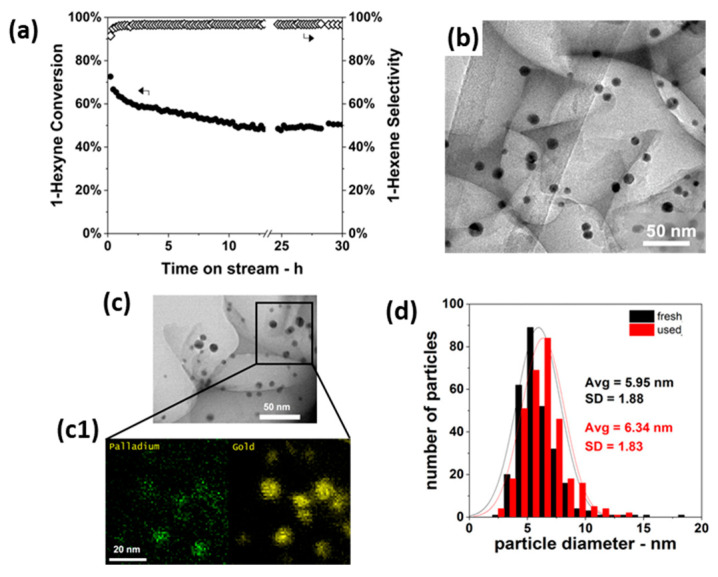
(**a**) 1-hexyne conversion and 1-hexene selectivities in 1-hexyne partial hydrogenation as a function of TOS for Pd_0.04_Au_0.96_ RCT-SiO_2_. Reaction conditions: T = 90 °C, P = 1 atm, 1% (*V*/*V*) 1-hexyne; 20% (*V*/*V*) H_2_, He balance, m_cat_ = 20 mg, total flow rate = 50 mL min^−1^, GHSV = 3800 h^−1^. (**b**) TEM of fresh Pd_0.04_Au_0.96_ RCT-SiO_2_. (**c**) TEM of Pd_0.04_Au_0.96_ RCT-SiO_2_ after the stability test in (**a**); (**c1**) STEM-EDX color mapping of (**c**); green: Pd; yellow: Au. (**d**) Particle size distributions of fresh and used Pd_0.04_Au_0.96_ RCT-SiO_2_. Adapted with permission from Ref. [182]. Copyright 2020 American Chemical Society.

Analogous to their previously mentioned application in CO oxidation [172], Luneau et al. [182] demonstrated the use of Pd-dilute PdAu RCT-SiO_2_-embedded nanocatalysts for the selective hydrogenation of 1-hexyn into 1-hexene. A Pd_0.04_Au_0.96_ catalyst, i.e., with optimized composition, proved superior to monometallic Pd as it achieved high alkene selectivities (>90%) at high alkyne conversions (~80%) for an extended reaction time of 30 h (Figure 12a). The authors attributed the selectivity results to the dilute surface concentration of Pd active sites, which would favor partial hydrogenation [183,184,185,186]. Comparative (S)TEM(-EDX) analysis, represented in Figure 12b–d, revealed neither sintering nor Pd-Au phase separation after calcination or reaction, thus demonstrating the stabilization effect of the catalyst’s architecture. While carbon formation was proved by temperature-programmed oxidation, no detrimental effects on catalytic performance were observed.

Stability improvement by embedding NPs is a promising tactic to further advance the industrial and environmental potential of catalysts for CO_2_ hydrogenation towards MeOH. A first successful synthesis of core-shell Cu@mesoporous SiO_2_ (m-SiO_2_) and Cu/ZnO@m-SiO_2_ catalysts was reported by Yang et al. [187]. Both Cu and Cu/ZnO NP centers conserved their monodisperse (~5 nm) size distribution after calcination, reduction, and reaction, as opposed to a reference Cu/m-SiO_2_ sample, where notable sintering occurred. As a result of the high metal dispersion in the core-shell architectures and their anti-sintering properties, these excelled in terms of conversion, MeOH selectivity and stability. Additionally, Cu/ZnO@m-SiO_2_ materials achieved twice the selectivities of monometallic Cu@m-SiO_2_ due to the introduction of additional basic sites by ZnO. 

As an alternative to the aforementioned Cu/ZnO materials, Shi et al. [188] synthesized CuIn@SiO_2_ core-shell materials with superior anti-segregation properties. In activity tests, the catalytic performance was superior to that of CuIn/SiO_2_, both initially and after 100 h TOS. These results were attributed to both the isolating nature of the shell as well as to the Cu-In interactions within the core promoting Cu dispersion, metal reducibility, alloy formation, and oxygen vacancy formation, and thereby CO_2_ activation and hydrogenation.

#### 2.4.3. Reforming Reactions

As hydrocarbon reforming is performed at high temperatures, one of the intrinsic challenges of these reactions is the rapid catalyst deactivation due to coke formation and sintering of the active phases. To counter these degradation effects, the design of embedded reforming catalysts has been studied extensively over the past decade. In view of the in-depth review on this topic written by Li et al. [162] at the end of 2018, only more recently published work will be covered in this section.

Among monometallic non-noble DRM catalysts, Ni materials are by far the most interesting as they exhibit higher activities compared to Co- or Fe-based DRM catalysts. In result, encapsulated Ni materials have been the subject of many studies [189]. Continuing from their previously reported colloidal protocol for the preparation of sinter-free and carbon-resistant core-shell-structured Ni@SiO_2_ [190], Zhang and co-workers [191] evaluated the effect of Ni NP size and MSI on the catalyst’s performance in DRM. To this end, catalysts with average Ni NP sizes of 1.4, 1.9 and 2.6 nm, as determined via TEM after activation, were prepared by employing calcination temperatures of 500, 600 and 700 °C, respectively. Reduction temperature analysis via H_2_-TPR indicated the strongest MSI occurred for higher calcination temperatures. The catalytic performance of the sample with intermediate NP size and intermediate MSI strength was superior in overall reforming activity and stability indicating that the DRM performance in these catalysts is both function of NP size and MSI. The group also demonstrated the stability of the same Ni@SiO_2_ catalyst in methane steam reforming (SRM) after 50 h TOS [192]. Additional TEM and XRD characterization revealed that the architecture conserved the initial morphology after SRM up to 750 °C. Similar structural and catalytic stabilities were observed when the catalyst was subjected to DRM at identical temperatures. However, the spent catalyst contained a higher carbon content after DRM than after SRM, which was attributed to the stronger carbon gasification power of H_2_O relative to CO_2_. 

#### 2.4.4. Water-Gas Shift 

As WGS is one of the prime reactions for H_2_ production [193], improved design of WGS catalysts is of high importance for industry. While Ni [194] is a cost-effective alternative to noble metal-based [195] catalysts, it suffers from two intrinsic drawbacks under WGS conditions [196]: simultaneous CH_4_ formation, which restricts the H_2_ yield; and sintering of Ni. As a solution to these problems, Gao et al. [197] synthesized core-shell hexagonal boron nitride (h-BN)-encapsulated Ni NPs through a facile thermal NH_3_ treatment of PVP-capped Ni NPs. These core-shell Ni@h-BN catalysts retained their particle size under thermal treatments up to 850 °C and showed high WGS activity at low temperatures (~250 °C). In a stability test, the catalyst’s CO conversion remained stable at 90–92% during 30 h TOS, indicating no deactivation by sintering or coke formation. In that time, very little CH_4_ formation was observed, proving the beneficial role of the h-BN shell in increasing the H_2_ yield. Based on activity data and H_2_O pulse chemisorption experiments, it was suggested that h-BN mitigates methanation by inducing H_2_O dissociation at the h-BN surface or at the core–shell interface.

## 3. ALD in Gas-Phase Catalysis

### 3.1. Principles of ALD for Supported Catalyst Preparation

Within the toolbox of techniques for controlled heterogeneous catalyst design, ALD [31,198], a vapor-phase method, is an interesting alternative to colloidal NP generation. ALD is a thin film deposition technique whereby uniform (sub)monolayers of metals, metal oxides, nitrides or sulfides are directly and sequentially deposited onto a substrate through cycles of self-limiting reactions between the gaseous chemical precursors and the substrate [198]. By self-limiting, it is meant that the surface growth reactions are terminated when one of the reagents, i.e., the functional groups on the substrate, is depleted and the surface is “self-saturated”. 

**Figure 13:**
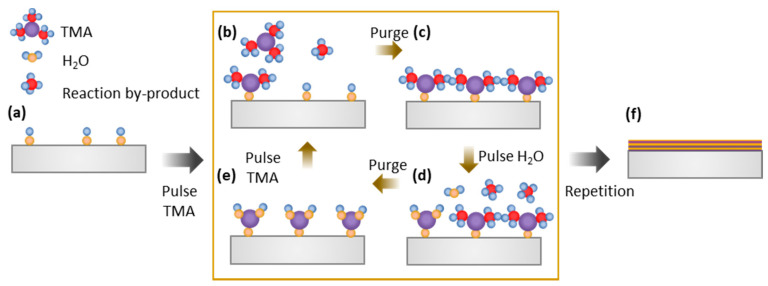
Schematic representation of an AB-type ALD process, illustrated for the deposition of Al_2_O_3_ films on a SiO_2_ substrate using a TMA/H_2_O ALD process. (**a**) SiO_2_ substrate surface with OH groups. (**b**) Pulsing vapor-phase TMA leads to a first surface reaction, with by-product formation. (**c**) When the surface is saturated, excess TMA and by-products are purged out. (**d**) The second reactant, H_2_O vapor, is pulsed and the second surface reaction takes place. (**e**) The substrate surface is saturated with H_2_O; a first alumina (sub-)monolayer is formed and H_2_O and by-products are purged. (**f**) Steps b–e are repeated to form an alumina film with the desired thickness. Grey: SiO_2_ substrate; orange sphere: oxygen atom; blue sphere: hydrogen atom; red sphere: carbon atom; purple sphere: aluminum atom.

To illustrate this self-limiting nature, the deposition of Al_2_O_3_ films on a SiO_2_ substrate using a trimethylaluminum (TMA)/H_2_O ALD process [199] is represented in Figure 13. In a first step, the substrate is exposed to a pulse of gaseous TMA, enabling a first surface reaction between the precursor molecules and the OH groups on all of the substrate’s exposed surfaces. When all substrate-related functional groups have reacted, the surface has reached its self-saturated state, such that additional reactions are inhibited. Hereafter, the precursor pulse is stopped and unreacted TMA as well as by-products formed from the surface reaction are purged by inert gas or under vacuum. In the following step, H_2_O vapor is pulsed into the reactor in view of the second self-limiting surface reaction, which brings the chemisorbed TMA through reaction with H_2_O to the desired phase deposition. This creates the first alumina (mono)layer with the same functional groups as the substrate material. After a second purge, the cycle can begin anew such that it is possible to deposit material one atomic layer at a time. It must be noted that, apart from the illustrative “AB” ALD process (A = TMA, B = H_2_O), also so-called “ABC processes”, which involve three compounds (and thus three self-limiting surface reactions followed by purging), are possible for the deposition of binary compounds as well as elemental films [200,201,202]. Furthermore, two ALD processes can be combined to obtain ternary phases or bimetallic materials, either as bilayers or stacked multilayers. A detailed description of possible ALD processes and the underlying reaction mechanisms is beyond the scope of this review. For a thorough description of this topic and the synthesis of catalysts via ALD in general, the reader is referred to the appropriate literature [28,30,31,32,39,200,201,202,203,204,205,206,207].

The self-limiting trait of ALD allows for the controlled deposition of conformal (even thickness over the exposed surface) films regardless of the substrate’s flatness, porosity, and surface area, making it, in principle, ideal for porous supports [208,209]. A prerequisite to achieve conformality in high surface area supports, and in particular mesoporous materials, is that the precursor molecules must be able to diffuse within the pores of the support material. It has been proved experimentally that the minimum pore diameter that can still be coated via ALD is determined by the size of the precursor molecule used. When the inner remaining pore diameter is on the same order of size as the precursor molecule, further deposition is no longer possible [29,210,211]. 

Even if the pore size is significantly larger than the size of the precursor molecule, the necessary contact between the precursor and support can be impeded by limitations in terms of diffusional mass transport and partial pressure of precursor and reactant at short exposure times. As such, long exposure times may be necessary to achieve surface saturation. On the other hand, thin layers of support (powder) can ensure diffusion of the precursor and reactant down to the bottom of the sample batch. Alternatively, dedicated ALD reactor setups [212] can be employed to shuffle the support material, e.g., vibrating cup, fluidized bed [213], or rotary reactor [214,215], thereby enabling contact between most of the surface area and gas-phase molecules.

While the description in Figure 13 relates to the deposition of material layers, ALD can also establish the deposition of NPs. Supported NPs can be obtained by making use of the nucleation-controlled growth mode of metal ALD processes, implemented by an appropriate choice of precursor, reactant, substrate and ALD conditions [216,217,218,219]. Herein, well-dispersed metal clusters or islands are initially formed due to the lack of sufficient chemisorption sites for the precursor on the substrate and the nature of metals to adopt a crystalline structure. The growth of these nuclei proceeds by surface diffusion phenomena and the stronger interaction of metal precursors with the pre-deposited metal rather than with the substrate [206]. In addition, NP formation can be induced by thermal sintering of ALD material films into (sub-)nanometer-scale agglomerates, in a subsequent thermal treatment, e.g., calcination or high-temperature reduction by H_2_ [220]. 

The nucleation-controlled growth of metals via ALD makes this technique attractive for the purpose of supported NP generation for catalytic applications. Furthermore, the “layer-by-layer” deposition can be used to control the metal loading at the (sub-)monolayer level and to tune the average NP size by the total number of ALD cycles applied. Moreover, by taking advantage of selective deposition of the target material, ALD can be used to exert spatial control over the nanostructures at the atomic level [33,39]. For instance, this selective deposition can be used to implement the synthesis of core-shell, embedded, selectively coated and alloyed NP materials [221,222,223]. 

The choice for ALD is further illustrated by its advantages over other synthesis approaches. As a vapor-deposition method, the use of solvents is not necessary, which is in strong contrast with wet chemical approaches such as colloidal synthesis [13]. For completeness, it must be noted that, although ALD has traditionally been defined as a gas-phase deposition technique, recently the principle of ALD with self-saturating surface reactions has been transferred to precursors dissolved in a liquid/solvent [224,225,226]. This “solution ALD” was able to coat deep pores in a conformal manner, and can offer novel opportunities concerning cost, scalability, and precursor selection, as it makes the need for volatile and thermally robust precursors less stringent. Furthermore, colloidal approaches mostly require an intermediate step to deposit or graft the pre-synthesized NPs onto the substrate [46], which is not the case in ALD as metal precursors are directly ‘deposited’ onto the surface through chemisorption [31]. Moreover, deposited colloids require removal of the stabilizing agent under thermal treatment, which can compromise the NP morphology [21]. As such agents are not employed in ALD, the aforementioned problem is non-existent for this technique. Still, an activation treatment may be required for ALD catalysts in case the deposited state of the material does not correspond with the material’s active state, e.g., metal oxide (deposited) versus zerovalent state (active). Therefore, morphological influences by thermal treatments may still play a role in ALD catalyst preparation. In the case of embedded NPs synthesized by colloidal templates, the creation of a surrounding metal oxide phase is usually implemented by sol-gel chemistry [25,174,180,227,228] or chemical vapor deposition (CVD) [229], which results in relatively thick (>1 nm) shells that can easily impede mass transfer. Due to the self-limiting nature of the involved surface reactions, oxide coatings prepared by ALD can be tailored at the atomic scale, such that thinner shells can be obtained which do not restrict mass transfer. However, it can be argued that ALD is technically more ‘complex’ in nature than colloidal synthesis protocols due to the equipment involved, as the latter typically require nothing but a hot plate with stirrer and inert gas feed, whereas specialized reactors are necessary for the former.

Altogether, ALD offers formidable opportunities for atomically controlled design of solid catalysts. Dedicated papers have been published on the synthesis of catalysts via this technique [28,30,31,32,37,38,39,203,204,205], though related activity studies are not as abundant. In what follows, the focus will go to recent progress in the application of ALD-prepared materials on porous supports to gas-phase catalysis. Analogous to the case of colloidal synthesis, a distinction is made between monometallic, bimetallic, and oxide-coated systems. 

### 3.2. Monometallic Supported Catalysts by ALD

Currently, an estimated 1000 ALD processes exist for the deposition of a wide array of elements [207]. Because of the level of control inherent to ALD, the technique has received much attention for the systematic investigation of supported NPs and optimization of the factors that influence their catalytic performance, e.g., NP size, NP shape, MSI, etc.

#### 3.2.1. Oxidation Reactions

One of the major merits of ALD is its ability to apply conformal coatings on a support without significantly altering the support’s pore size and surface area [29]. For the purpose of preventing changes in the catalyst’s surface texture of Pd/γ-Al_2_O_3_ catalysts, Mao et al. [230] applied ALD to investigate the effect of interaction between promoter oxides and Pd on its performance in CO and CH_4_ oxidation. To this end, catalysts with well-defined promoter layers were prepared in two steps: (1) promotion of a γ-Al_2_O_3_ support through ALD deposition of a uniform NiO, Co_3_O_4_, Fe_2_O_3_, MnO_2_, CeO_2_, or ZrO_2_ film; and (2) Pd ALD onto the promoted supports. Compared to unpromoted Pd/γ-Al_2_O_3_, CO oxidation activity was significantly improved for CeO_2_-, Fe_2_O_3_- and MnO_2_-promoted supports, with the most notable increase for CeO_2_. No promotional effect was observed for all other oxides. These results were attributed to the reaction taking place at metal oxide-Pd interfaces, where oxygen species are supplied from the reducible metal oxides to Pd. This effect was more pronounced for Fe_2_O_3_, MnO_2_ and CeO_2_ due to the lower reducibility of these species compared to NiO, Co_3_O_4_ and ZrO_2_. The superior effect of CeO_2_ was ascribed to strong CeO_2_-Pd interactions. However, the contrary held true for CH_4_ oxidation, wherein only NiO and Co_3_O_4_ promoted the oxidation reaction, which is in accord with previous reports [231,232,233]. The underlying reason for the different degree of promotion for each oxide phase remains unclear.

**Figure 14:**
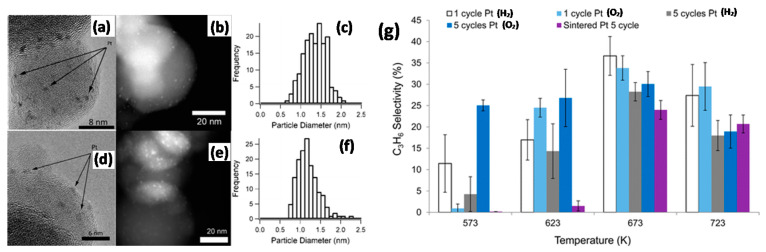
High-resolution TEM, HAADF-STEM and corresponding particle size distributions of Pt/Al_2_O_3_ catalysts prepared via (**a**)–(**c**) one Pt ALD cycle with O_2_, and (**e**)–(**f**) one Pt ALD cycle with H_2_. Arrows indicate Pt NPs. (**g**) Propylene selectivities as a function of temperature in the PODH reaction for Pt/Al_2_O_3_ catalysts prepared via Pt-H_2_/O_2_ ALD with a variable number of cycles. “Sintered Pt 5 cycles” denotes an ALD catalyst prepared using 5 Pt ALD cycles (O_2_) which was intentionally sintered to 1.6 nm (by treating in 100% H_2_ for 4 h at 600 °C). Reaction conditions: 12 sccm C_3_H_8_, 6 sccm O_2_, 182 sccm Ar, m_cat_ = 100 mg. Adapted with permission from Ref. [234]. Copyright 2015 American Chemical Society.

ALD has also been utilized to tune the Pt NP size of propane oxidative dehydrogenation (PODH) catalysts [234] in order to enhance their industrial relevance. Given the fact that PODH is a structure-sensitive reaction, ALD has much potential in creating catalysts with optimized nanostructures, and consequently maximum propylene selectivity. A first study of Pt/Al_2_O_3_ PODH catalysts synthesized by ALD was performed by Gould et al. [234], who prepared uniform NPs in the size range 1.0–2.5 nm by varying the number of Pt cycles and adjusting the type of reactant used. For a given number of Pt ALD cycles, the use of H_2_ led to smaller NPs, which is illustrated in Figure 14a–f for the case of 1 ALD cycle. Analogous effects of H_2_ and O_2_ reactants were obtained for 5 ALD cycles. In the more standard O_2_-based Pt ALD process, OH sites on the Al_2_O_3_ surface can serve as reaction sites for the Pt precursor, and the O_2_ reactant step results in ligand removal and adsorbed O on the deposited Pt surface atoms. The latter can react with the precursor during the next step of the ALD process. In contrast, in situ IR spectroscopy during ALD-related H_2_ treatment pointed towards the depletion of surface OH groups on the Al_2_O_3_ support [234]. Moreover, a metallic Pt surface will be formed in this case. Both these effects slow down the adsorption of precursor molecules, possibly explaining the reduced Pt NP growth for the H_2_-based process [234]. Differences in surface diffusion mechanisms may also explain the different nucleation behavior during these Pt ALD processes [219,235,236]. At optimized conditions, the catalysts with the lowest average size, i.e., 1 cycle Pt ALD with H_2_, achieved the highest activity, with a maximum C_3_H_6_ TOF of ~0.42 s^−1^, and propylene selectivity of 37% (Figure 14g). A commercial Pt/Al_2_O_3_ catalysts (average NP size 3.6 nm), on the other hand, achieved propylene selectivities lower than 1% under the same conditions. 

#### 3.2.2. Hydrogenation Reactions

To study the promotional effect of Pd-TiO_2_ interactions on the selective hydrogenation of acetylene over Pd catalysts, Gong and co-workers [237] prepared Pd/TiO_2_/MCM-41 via successive ALD of TiO_2_ and Pd on MCM-41. For comparison, similar materials were synthesized by Pd IWI of ALD TiO_2_-modified MCM-41. The performance of “control ALD catalysts” was superior to that of catalysts prepared via IWI. This was attributed to the more uniform and well-dispersed nature of the ALD-prepared Pd NPs in the mesopores of MCM-41. These mesopores exhibit a space confining effect on the NPs, which helps maintain the well-dispersed and uniform morphology of the as-prepared material and hence the number of available surface sites during reaction. In addition, MSI between TiO_2_ and Pd, created by subjecting the catalyst to a reductive treatment after ALD, were found to limit the adsorption of acetylene and ethylene, resulting in an increase in ethylene selectivity relative to an unpromoted Pd/MCM-41 sample. For stronger MSI, induced when the ALD sample was subjected to reductive treatments at higher temperatures, catalyst deactivation by carbon formation was less pronounced in stability tests. 

Kim and co-workers [238] applied ALD as a means to synthesize well-defined Pt nanoclusters (<1 nm) on NU-1000, a metal-organic framework (MOF), for their application in ethylene hydrogenation. Their choice of support was motivated by its high surface area, thermal and chemical stability, the presence of -OH and -OH_2_ functionalities within the mesoporous (~3 nm) channels—which makes it ideal for ALD—and the space-constraining effect of the framework’s organic linker components—which mitigates metal atom migration. Under steady-state conditions, Pt/NU-1000 materials proved highly active and stable after ~20 h TOS. Moreover, infrared (IR), differential X-ray pair distribution function (PDF) and EXAFS analyses evidenced the catalyst’s sintering resistance up to 200 °C in inert, H_2_ and ethylene hydrogenation atmospheres.

With the purpose of upscaling non-noble nanocatalyst design for hydrogenolysis purposes, Gould et al. [239] reported the use of an AB-type ALD process to deposit metallic Ni on an Al_2_O_3_ substrate. Propylene was used in their case study to differentiate the catalyst’s role in the hydrogenolysis and hydrogenation of this molecule. By varying the number of cycles between 1 and 15, Ni loadings and average Ni NP sizes in respective ranges of 4.7–16.8 wt% and 2.4–3.3 nm could be obtained. Increasing the number of ALD cycles decreased the dispersion from ~51% to ~37%. These dispersion values were superior to those of catalysts prepared via IWI and WI methods, as these amounted respectively to 6.5% and 3.6%. Additionally, the ALD-prepared samples differed from these reference samples in terms of surface structure as CO temperature-programmed desorption unveiled a threefold increase in desorption from kinks and step sites compared to (I)WI catalysts. Since hydrogenolysis is more favorable over such step and kink sites [240,241], the hydrogenolysis selectivities of ALD Ni/Al_2_O_3_ catalysts were much higher, attaining a maximum of 10.4% for the 15-cycle catalyst at 250 °C, whereas the selectivity of the impregnated samples was below 0.4% at the same temperature.

#### 3.2.3. Reforming Reactions

One of the biggest challenges for Ni reforming catalysts is coking. It has been established that the use of small Ni NPs can circumvent this problem, as such NPs cannot support carbon nucleation and growth [242]. In that regard, the small and monodisperse particle sizes that can be obtained through ALD make this technique ideal for the design of coke-resistant reforming catalysts. Furthermore, since ALD is based on chemisorption, ALD-prepared materials usually exhibit strong interactions between the active phase and the support, which mitigates metal sintering. 

**Figure 15:**
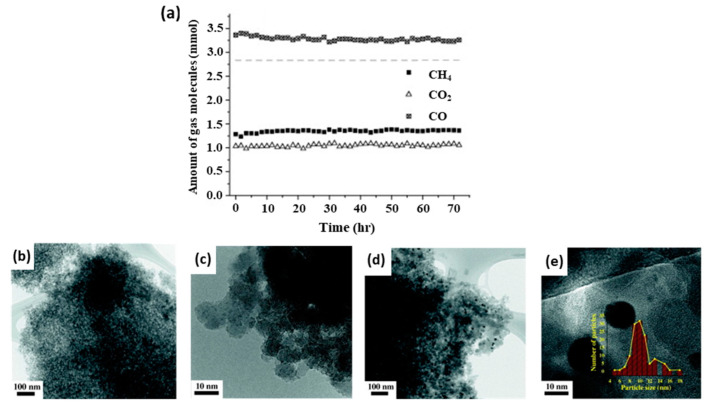
(**a**) Amount of gas molecules as a function of time in the effluent of a reactor containing a 50Ni/m-SiO_2_ ALD catalyst subjected to DRM at 800 °C. Reaction conditions: T = 800 °C, P = 1 atm, CH_4_/CO_2_ = 1/1 (*V*/*V*), total flow rate = 20 mL min^−1^, m_cat_ = 100 mg. (**b**) TEM image of 50Ni/m-SiO_2_ before DRM and (**c**) corresponding high magnification TEM image. (**d**) TEM image of 50Ni/m-SiO_2_ after DRM and (**e**) corresponding high magnification TEM image. The inset in (**e**) is the Ni NP size distribution of the sample in (**d**). Reprinted with permission from Ref. [243]. Copyright 2013 Elsevier.

To our knowledge, the first report of such stable ALD Ni catalysts was issued by Kim et al. [243]. In their work, Ni/m-SiO_2_ was prepared by 50 cycles of Ni ALD on mesoporous SiO_2_ (50Ni/m-SiO_2_). The catalyst achieved high initial activities and remained highly stable for 72 h TOS in DRM at 800 °C (Figure 15a). This stability was corroborated by limited metal sintering and coke formation observed via TEM (Figure 15b–e), XPS and XRD. As the performance of unsupported Ni NPs was inferior, the excellent performance of ALD Ni/m-SiO_2_ was attributed to a strong interaction between the metal and support phase and a partial embedment of the NPs within the m-SiO_2_ phase. 

In other work, Shang et al. [244] applied Ni ALD to porous γ-Al_2_O_3_. Apart from metallic Ni NP formation (average size 3.6 nm), Ni deposition resulted predominantly in the formation of NiAl_2_O_4_, which was confirmed by XPS of the catalyst after ALD. This NiAl_2_O_4_ phase could not be reduced by H_2_ up to 700 °C, i.e., the initially used activation step in their study. However, NiAl_2_O_4_ did reduce into active Ni^0^ under DRM conditions at 850 °C. As such, the material proved largely inactive in DRM after reduction by H_2_, yet after ‘DRM activation’, the catalyst’s performance was exceptional (maximum 93% CH_4_ conversion at 850 °C). Moreover, when subjected to three DRM cycles in the temperature range 700–850 °C, it maintained excellent activity, indicating minimal deactivation by coking or sintering. Analogous to the work of Kim et al. [243], this stability was related to the strong interaction between Ni and Al_2_O_3_ as well as to the support’s highly porous structure promoting these interactions. The role of porosity was supported by the fact that the use of dense γ-Al_2_O_3_ NPs as support yielded catalysts with activity and stability inferior to that of porous γ-Al_2_O_3_. Similar observations of NiAl_2_O_4_ formation and exquisite stability in DRM were observed when the same group applied their Ni ALD process to hollow α-Al_2_O_3_ fibers [245]. Again, the high stability was attributed to strong Ni-support interactions. 

Stable ALD Ni/γ-Al_2_O_3_ DRM catalysts have also been reported by Wang et al. [246]. Contrary to the results of Shang and co-workers [244], they ascribed the stability to the presence of easily reducible NiO in the as-prepared state, rather than to NiAl_2_O_4_, due to weaker Ni-Al_2_O_3_ interactions. The authors ascribed this to the different type of γ-Al_2_O_3_ support used in the work of Shang et al. [244]. Because of the presence of the NiO phase, the Ni/γ-Al_2_O_3_ material of Wang et al. already proved active in DRM after regular reduction by H_2_ at 700 °C. After 35 h TOS, minimal deactivation occurred. This was attributed to insignificant Ni sintering due to the moderate MSI, as well as limited nucleation and growth of graphitic carbon on the small (~3–4 nm) Ni NPs. Of all examined ALD catalysts (0.8, 1.6 and 2.0 wt% Ni), the catalytic performance was optimal for a 1.6 wt% Ni material. The initial activities of ALD catalysts were higher than those of similar materials prepared via a WI route. Moreover, significant sintering and coke formation were observed, which was linked to the larger, less-dispersed nature of the Ni NPs created via conventional WI. The enhanced performance of ALD-prepared DRM catalysts due to smaller NP sizes relative to those prepared via WI, has also been confirmed for Rh/γ-Al_2_O_3_ [247]. 

Similar Ni ALD methods have been applied in the development of n-dodecane steam reforming catalysts by Li et al. [248]. They reported Ni/γ-Al_2_O_3_ with a fourfold increase in TOF values compared to a WI-prepared catalyst, due to relatively weaker Ni-Al_2_O_3_ interactions that increase reducibility and NP dispersion. However, the weak MSI induced notable sintering, albeit to a lesser degree than the WI material. Increasing the strength of MSI through the addition of CeO_2_ mitigated this problem.

### 3.3. Bimetallic Supported Catalysts by ALD

ALD processes can be extended to deposit two different metallic precursors onto a support. As stated in Section 3.1, this is established through the combination of two ALD processes. Due to the self-limiting nature of ALD, this allows control over the catalyst’s NP size, composition, and architecture at the atomic level:

- the NP size can be tailored by adjusting the total number of ALD cycles;
- the relative number of ALD cycles of each element determines the composition;
- the order of deposition determines the NP architecture.

The added benefits of combining two metals, as discussed in Section 2.3, along with the aforementioned level of control enabled by ALD, make the fabrication of such supported bimetallic catalysts via this technique very interesting for activity studies. Most literature on the application of bimetallic catalysts prepared by ALD is focused on materials containing only noble metals [31,32,205,222,249,250,251,252]. Nevertheless, successful extensions to systems containing non-noble metals have been reported. To avoid the repetition of prior reviews [32,39,205] and accredit the economic importance of non-noble metal-containing materials, only these are covered in this section.

#### 3.3.1. Dehydrogenation Reactions

**Figure 16:**
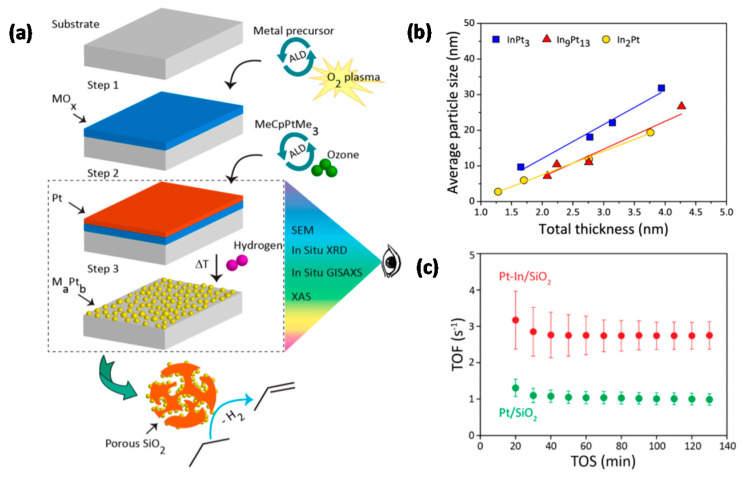
(**a**) Schematic representation of an ALD method to synthesize bimetallic PtM NPs (where M = In or Ga) on planar or porous SiO_2_: (step 1) MO_x_ layer deposition via ALD, (step 2) deposition of a Pt layer via ALD and (step 3) conversion of the bilayer into PtM NPs via H_2_-TPR. In the same study, SEM, XAS, in situ XRD and GISAXS were applied to examine the bilayer-to-NP conversion. (**b**) Average particle size (determined by SEM analysis after TPR in 10% (*V*/*V*) H_2_/N_2_ up to 700 °C) as a function of total layer thickness for various PtIn phases prepared by the bilayer method. Solid lines are linear trends fitted to the experimental data. (**c**) TOF values for propylene production as a function of TOS for ALD-prepared InPt_3_/SiO_2_ and Pt/SiO_2_ in PDH at 600 °C. Reaction conditions: W_cat_/F_C3H8,0_ = 20 kg_cat_ s mol^–1^ and P_C3H8,0_ = 20 kPa, total pressure = 101.3 kPa. Adapted with permission from Ref. [253]. Copyright 2016 American Chemical Society.

Within the scope of gas-phase catalysis, most non-noble metal-containing materials prepared via ALD have been developed and applied for dehydrogenation reactions. Ramachandran et al. [253] developed a bilayer approach to tailor the size and composition of Ga- and In-promoted Pt-catalysts for PDH purposes. Herein, PtM (where M = In or Ga) alloy NPs were deposited onto a planar SiO_2_ substrate in a three-step process, shown in Figure 16a. Specifically for In, size control in the range 1–30 nm was obtained by varying the total bilayer thickness via the total number of ALD cycles (Figure 16b), while the particle composition could be tuned over the entire Pt-In compositional range by varying the relative thickness of each metal’s layer, i.e., the relative number of ALD cycles for each metal. The formation of PtIn nanoalloy particles, induced by high-temperature reduction by H_2_ of the Pt/In_2_O_3_ bilayer, was evidenced by in situ XRD, in situ grazing incidence small-angle X-ray scattering (GISAXS), XAS and electron microscopy. When applied to a porous SiO_2_ support, such ALD InPt_3_/SiO_2_ catalysts exhibited activities (Figure 16c) and propylene selectivities similar to other state-of-the-art PDH catalysts [254,255]. Though deactivation via coking occurred, the initial catalytic activity could be restored after coke removal and regeneration via oxidation-reduction cycles. Both in situ GISAXS and in situ XRD confirmed realloying and restoration of the morphology after these regeneration cycles.

In the context of 1,3-butadiene (BDE) production via the non-oxidative dehydrogenation of butane, Camacho-Bunquin and co-workers [256] developed an ALD-based strategy to fabricate uniform, well-dispersed, SiO_2_-supported PtZn NPs with sizes of 1.3 ± 0.3 nm. Under optimized dehydrogenation conditions, these PtZn/SiO_2_ catalysts exhibited a higher BDE yield (9.1–9.7%), activity and coke resistance relative to ALD-prepared monometallic Zn/SiO_2_ and an alternative PtSn/SiO_2_ catalyst prepared via a WI method. The superior catalytic performance of PtZn/SiO_2_ was attributed to the relatively smaller NP sizes, the high dispersion of PtZn clusters and the positive promotional effect of Zn on Pt.

**Figure 17:**
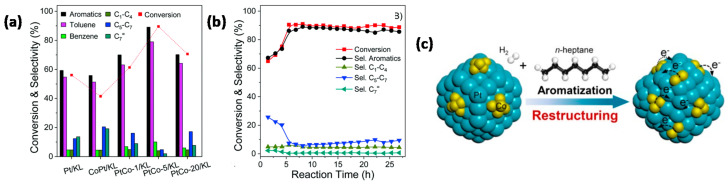
(**a**) Conversion of *n*-heptane and product selectivities for ALD catalysts in the aromatization of *n*-heptane measured after 5 h. “PtCo/KL” denotes Pt ALD on a KL zeolite followed by Co ALD, and “CoPt/KL” vice versa. “PtCo-n” (*n* = 1, 5 or 20) denotes a catalyst with 5 Pt ALD cycles and n Co ALD cycles. (**b**) Conversion of *n*-heptane and product selectivities as a function of time for PtCo-5/KL. Reaction conditions for (a) and (b): T = 420 °C, P = 1 atm, H_2_/*n*-heptane = 6 (*V*/*V*), WHSV = 0.68 h^−1^. (**c**) Representation of the restructuring of PtCo clusters induced by the aromatization of *n*-heptane. Reproduced from Ref. [257] with permission from the Royal Society of Chemistry.

Wang et al. [257] prepared *n*-heptane aromatization catalysts with fine control over the catalyst’s structural properties through the sequential deposition of Pt and Co onto a KL zeolite, for investigating the promotional effect of Co on Pt. Optimized “PtCo-5/KL”, i.e., with 5 Pt and 5 subsequent Co ALD cycles, achieved the highest aromatic selectivities (Figure 17a), as well as activities that were higher by a factor 1.6 than for monometallic Pt/KL. The promotional effect of Co was further illustrated by the catalyst’s stability in a 25 h TOS test (Figure 17b), wherein the *n*-heptane conversion and aromatic selectivity respectively increased from 62% to 90% and 67% to 89.1% within the first 5 h and then remained at these final values. This performance enhancement after a 5-h induction period was linked to the restructuring of the catalyst under the reactive environment within this time period, represented in Figure 17c. Herein, the initial Co clusters on the Pt NPs migrate into smaller Co clusters. This rearrangement introduces an electron-donating effect from Co towards Pt, which in turn facilitates the electron transfer from Pt to *n*-heptane, thereby promoting the dehydrocyclization of *n*-heptane. DFT calculations associated the catalytic stability of the restructured bimetallic system with its thermodynamic stability.

#### 3.3.2. Reforming Reactions

**Figure 18:**
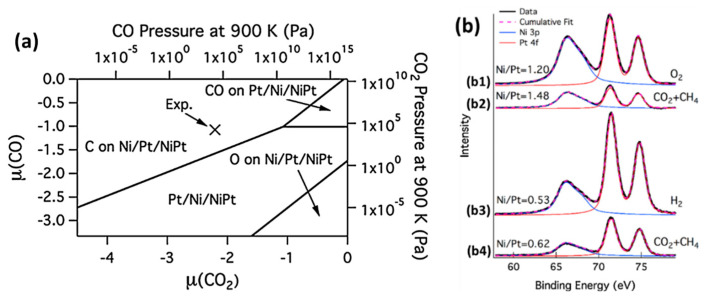
(**a**) Bimetallic NiPt surface phase diagram as a function of the chemical potentials of CO (μ(CO)) and CO_2_ (μ(CO_2_)). “X” represents chemical potentials under true reactive DRM conditions, assuming atmospheric total pressure. (**b**) NAP-XPS spectra for a Ni-Pt crystalline surface (**b1**) exposed to O_2_ (4.3 × 10^−5^ Pa pressure) for 30 min at 227 °C; (**b2**) subsequently annealed up to 250 °C in an equimolar mixture of CO_2_ and CH_4_ (P_tot_ = 130 Torr); (**b3**) subsequently annealed to 400 °C in H_2_ (4.3 × 10^−5^ Pa pressure); and (**b4**) subsequently annealed to 300 °C in an equimolar mixture of CO_2_ and CH_4_ (130 Torr total pressure). Reprinted with permission from Ref. [262]. Copyright 2015 Elsevier.

In the class of Ni-based DRM catalysts, the addition of Pt is an established route to increase catalytic activity and stability [258,259,260,261]. The effects of Pt addition and catalyst preparation method were investigated by Gould and co-workers [262], who prepared NiPt/γ-Al_2_O_3_ via ALD and IWI routes and compared the bimetallic catalysts’ performance to ALD- and IWI-prepared Ni/γ-Al_2_O_3_. Due to the coke-inhibiting effects of the ~8 nm smaller nanostructures in ALD materials (relative to IWI samples), both mono- and bimetallic ALD catalysts performed better compared to IWI catalysts in terms of activity and resistance against coking. Relative to ALD Ni/γ-Al_2_O_3_, ALD NiPt/γ-Al_2_O_3_ was about two times more active, with reforming rate values exceeding those of other reported NiPt materials [259,260,261]. This improved DRM performance was attributed to synergistic Ni-Pt interactions. First principle calculations (Figure 18a) indicated that, under DRM conditions, the NiPt catalyst surface forms a Ni-terminated alloy structure with improved DRM formation rate over its monometallic counterparts. NAP-XPS (Figure 18b) confirmed the formation of such Ni-terminated phases. Additionally, it was proposed that Pt “defects” on the surface may increase the atomic carbon diffusion barrier, explaining the overall reduced coke formation on the bimetallic system.

### 3.4. Oxide-Coated Catalysts by ALD

ALD of metal oxide coatings, whereby a uniform and conformal metal oxide layer is deposited on a pre-made catalyst, has been extensively applied to improve catalytic performance [28,31,32,39,205]. The choice for ALD over other techniques for oxide deposition, e.g., CVD [229] or sol-gel chemistry approaches [174,180,227,228], is mainly motivated by its atomic-level control over the oxide layer’s thickness and composition [29,263,264,265]. This allows engineering coated systems with high tunability and with minimal mass transfer limitations. Another advantage of ALD coatings lies in the fact that these can be applied to a solid catalyst prepared by any synthesis technique, e.g., (I)WI, colloidal synthesis, ALD, ion exchange or DP. This is in major contrast to the techniques in Section 2.4, which are limited to the ‘deposition’ of oxides on colloidal NPs. Among the functions of an oxide coating, its stabilizing role by acting as a physical barrier against metal sintering is the most obvious [266,267,268]. Apart from inhibiting metal mobility, ALD-deposited (sub-)nanometer oxide layers can introduce additional metal-oxide interfaces, which play an active role in a wide variety of reactions, e.g., CO oxidation [230] and MeOH synthesis [269]. As such, the deposition of an extra oxide layer can have a promotional effect on the catalyst’s activity. Moreover, oxide layers can be deposited selectively on certain surface sites through so-called “site-selective ALD” (see also Section 3.5), leaving only other surface sites to participate in a catalytic reaction [270,271,272]. In the case of structure-sensitive reactions, such selective metal oxide ALD allows enhancing product selectivity.

While ALD oxide coatings provide many functionalities for tuning the catalytic performance, it is of course crucial that the NP surface remains accessible for reaction. In the case of ALD coatings, this instigates the need for ultra-thin coatings, which are not yet fully closed, or the spontaneous occurrence of cracks in the thin ceramic coating, e.g., triggered by mismatches in thermal expansion between the coating and the underlying material. An alternative approach consists in the deposition of a microporous ceramic coating, which can enclose the catalyst particle, but where the porosity may still allow access to the catalyst surface. An interesting approach to achieve this is offered by molecular layer deposition (MLD) [273,274,275,276], which is a variant of ALD but aims at the conformal deposition of hybrid coatings that contain both organic as well as inorganic fragments. An example is MLD of “metalcones” [275,277,278,279,280,281], which are hybrid coatings deposited by combining ALD precursors with alcohols as reactant. MLD inherits the conformality and atomic level thickness control from ALD, and therefore can be used to ‘envelop’ a catalyst particle. After MLD, a calcination step can be used to remove the organic fragments from the hybrid coating, which can result in a microporous shell [282,283,284].

Excellent reviews have been published on the topic of ALD coatings to improve catalytic stability, activity, and selectivity [28,31,32,39,205]. In this section, an overview is given of recent applications of such coatings in reaction studies. For a more extensive overview including older examples, reference is made to the aforementioned reviews.

#### 3.4.1. Oxidation Reactions

In recent work, ALD oxide coatings have been employed in the design of highly active and sintering-resistant CH_4_ oxidation catalysts. Cui et al. [285] applied Al_2_O_3_ ALD cycles to hydrophobic Al_2_O_3_-supported Pd (Pd/H-Al_2_O_3_). Bare Pd/H-Al_2_O_3_ suffered from significant deactivation after aging (air, 700 °C, 4 h) due to Pd NP sintering (7.5 to 13 nm). However, depositing 45 Al_2_O_3_ layers improved the thermal stability by mitigating sintering; pre- and post-reaction average Pd NP values amounted to 9.5 and 11 nm, respectively. In addition to Al_2_O_3_, ZrO_2_ modification has been demonstrated to have a stabilizing effect on CH_4_ oxidation catalysts. In the work of Onn and co-workers [286], ZrO_2_/Pd/CeO_2_ maintained its initial crystallite size and surface area after calcination in air at 400–800 °C, whereas unprotected Pd/CeO_2_ did not. Moreover, for higher calcination temperatures, ZrO_2_/Pd/CeO_2_ exhibited higher oxidation activities than Pd/CeO_2_. The authors attributed this to the oxidation-promoting effect of ZrO_2_ [287,288,289,290]. When exposed to a SO_2_-rich reagent stream, the oxide film proved an effective chemical barrier against SO_2_ poisoning. Apart from CH_4_ oxidation, the catalyst proved active for WGS, wherein the ZrO_2_ film also exhibited a stabilizing, activity enhancing, and poison-resistant role.

**Figure 19:**
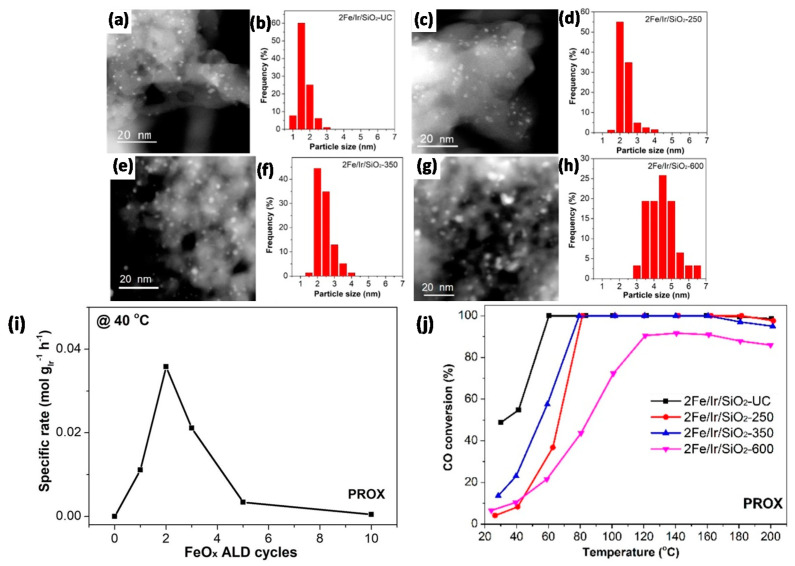
HAADF-STEM and corresponding particle size distributions for (**a**)–(**b**) uncalcined 2-Fe/Ir/SiO_2_ ALD catalysts (2-Fe/Ir/SiO_2_-UC), and calcined in air at (**c**)–(**d**) 250 °C (2-Fe/Ir/SiO_2_-250), (**e**)–(**f**) 350 °C (2-Fe/Ir/SiO_2_-350) and (**g**)–(**h**) 600 °C (2-Fe/Ir/SiO_2_-600). (**i**) Specific rate of Fe/Ir/SiO_2_-350 catalysts in the PROX reaction at 40 °C as a function of the number of applied FeO_x_ ALD cycles. (**j**) CO conversion as a function of temperature for 2Fe/Ir/SiO_2_ catalysts in the PROX reaction. Reaction condition for (**i**) and (**j**): 1% (*V*/*V*) CO, 1% (*V*/*V*) O_2_, 48% (*V*/*V*) H_2_, Ar balance. WHSV = 18,000 mL h^−1^ g_cat_^−1^. Adapted with permission from Ref. [295]. Copyright 2019 American Chemical Society.

Besides CH_4_ oxidation, the preferential oxidation of CO in H_2_ (PROX) [291] can benefit from the deposition of oxide coatings. PROX finds its use in hydrogen fuel cell applications, wherein residual CO contents in the H_2_ fuel stream, e.g., stemming from hydrocarbon reforming processes [193], must be removed as to avoid CO poisoning of the cell’s electrodes [292]. It has been proved [293,294] that FeO_x_-Ir interfaces have a promotional effect on the selective CO oxidation activity. To tailor these interfacial sites, Wang et al. [295] developed a protocol for the selective deposition of FeO_x_ via ALD on the Ir NPs of Ir/SiO_2_. Average NP sizes increased when the catalysts were subjected to calcination in air, with higher calcination temperatures resulting in larger sizes (Figure 19a–h). Optimal PROX activity was found when 2 cycles of FeO_x_ were deposited (Figure 19i) and for the smallest NP sizes (1.5 ± 0.6 nm), i.e., an uncalcined 2Fe/Ir/SiO_2_ material (Figure 19j). This was correlated with an optimal amount of PROX-active FeO_x_-Ir interfacial sites. As shown in Figure 19j, 2Fe/Ir/SiO_2_ exhibited 100% CO conversion over the temperature range 60–180 °C, which is one of the broadest ranges recorded for Ir catalysts to-date. This catalyst maintained 100% CO conversion and 50% selectivity during a 20 h TOS stability test at 80 °C. Interestingly, the activity towards CO oxidation in the absence of H_2_ was less pronounced, from which it was suggested that the presence of hydroxyl groups on FeO_x_ might play an important role in the PROX reaction.

#### 3.4.2. Hydrogenation Reactions

Within the framework of hydrogenation reactions, recent applications of ALD have focused on the study of promotional elements without introducing significant changes in the catalyst’s morphology. Asundi et al. [296] performed a fundamental study of MoO_3_ promotion of Rh/SiO_2_ catalysts for the selective conversion of syngas to alcohols. MoO_3_/Rh/SiO_2_ exhibited a ~66-fold increase in TOF values compared to unpromoted catalysts and MeOH selectivities up to 36%. Combined theoretical and in situ experimental studies linked this promotional effect to the presence of Mo-OH species substituted into the Rh NP surface. Based on DFT calculations, it was suggested that the presence of such Mo-OH species offers a novel hydrogenation pathway with low CO dissociation energy as well as a hydrogen spillover mechanism from Mo to Rh species, which together favor the selective CO hydrogenation towards MeOH with improved reaction rate.

Gao and co-workers [297] applied metal oxide ALD to elucidate the interactions between Cu and ZnO in methanol synthesis via CO_2_ hydrogenation. Both components of the active Cu/ZnO phase were brought together by applying ZnO ALD overlayers to Cu/SiO_2_. These materials exhibited high CO formation, yet limited MeOH formation rates and selectivities. From this performance, it was proposed that the right contact between ZnO and Cu^0^ is necessary to form MeOH; CO_2_ activates on ZnO, but requires the presence of hydrogen activated on neighboring Cu^0^ sites. A catalyst coated with 1 ZnO cycle of 30 s pulse time provided notable Cu^0^ metallic sites and synergy with the ZnO phase, such that MeOH selectivities of 10.1% could be obtained via this synthesis method

#### 3.4.3. Reforming Reactions

Increasing the thermal stability by depositing oxide overlayers via ALD is a route that has also been applied successfully for Ni-based DRM catalysts. In recent progress, Zhao and co-workers [298] applied alumina coatings to Ni/Al_2_O_3_, which was prepared via a WI approach, to synthesize a novel Al_2_O_3_/Ni/γ-Al_2_O_3_ “sandwich” material. Under DRM at 800 °C, an optimized material with 80 Al_2_O_3_ layers achieved initial CH_4_ and CO_2_ conversions of respectively 92% and 94%. After 400 h TOS, these values remained largely invariant. Post-reaction TEM imaging and TGA did not indicate any notable sintering nor carbon formation. These results were attributed to the small Ni NP size (~5 nm) in the initial catalyst preventing carbon nucleation, as well as the “dual MSI” on both the γ-Al_2_O_3_ support and the Al_2_O_3_ coating, which prevented sintering and consequential carbon formation.

**Figure 20:**
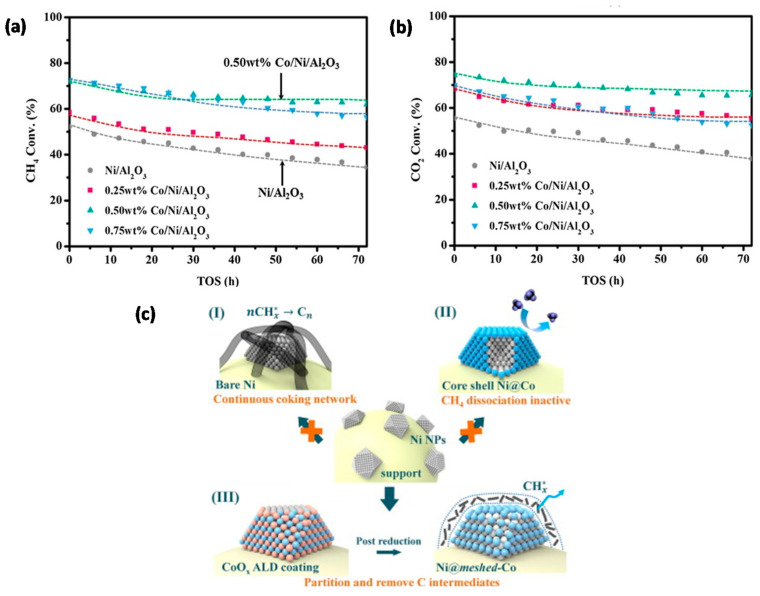
(**a**)–(**b**) DRM stability test results of Ni/γ-Al_2_O_3_ and meshed Co-coated Ni/γ-Al_2_O_3_ with 0.25, 0.50 and 0.75 wt% Co. Reaction conditions for (**a**) and (**b**): T = 650 °C, P = 1 atm, CH_4_/CO_2_ = 1/1 (*V/V*) with flow rate 60 sccm, m_cat_ = 300 mg. (**c**) Structure-property mechanism proposed for Ni- and CoNi/γ-Al_2_O_3_ catalysts for DRM. (I) Bare Ni/γ-Al_2_O_3_ suffers from coking via carbon nanotube formation originating from CH_4_ scission and CH_x_ intermediary accumulation. (II) A Ni@Co core-shell structure, prepared via excess CoO_x_ ALD cycles, is less active in DRM than Ni due to the dominant surface presence of Co. (III) A meshed Co coating on Ni (Ni@meshed-Co), prepared via CoO_x_ ALD coating of Ni/γ-Al_2_O_3_ and a subsequent reduction step, has enhanced DRM activity and coke resistance. Separation of Ni sites avoids a continuous carbon network generation and Co-Ni interfaces enhance carbon removal. Reprinted with permission from Ref. [299]. Copyright 2019 Elsevier.

Cao et al. [299] decorated Ni/γ-Al_2_O_3_ with sub-nanometer Co meshes, which increased the DRM activity and largely eliminated carbon formation and metal sintering. An optimized performance was achieved for a molar Ni/Co ratio of 12/1, whereby 0.50 wt% Co was deposited via six CoO_x_ ALD cycles (Figure 20a,b). Catalytic activity and stability improvements were ascribed to the meshed structure of the overlayer, illustrated in Figure 20c. The latter separates Ni sites, thus avoiding the formation of a continuous carbon network, typical for bare Ni; in addition, it confines Ni species, preventing them from sintering up to temperatures of 850 °C. Further, the presence of Co helps stabilize the metallic Ni state under DRM conditions, and finally, Co-Ni interfaces inhibit carbon formation and facilitate carbon removal. In all of the above, the meshed structure was essential. In the case of a Ni@Co core-shell structure, formed by excessive CoO_x_ ALD cycles, Co covers all Ni sites. As Co is less active in CH_4_ dissociation than Ni [300,301,302,303], this core-shell structure results in lower DRM activities than a meshed overlayer structure.

### 3.5. Area-Selective ALD for Next-Level Catalyst Design

Different crystallite facets, e.g., (111) or (100), and the active sites that accompany them (e.g., edge/terrace/corner), can yield different activity and product selectivity [304,305,306]. If control over these sites is possible, researchers are given an extra tool to put these sites selectively to use. To accomplish this, investigations are needed to acquire insight into site-specific activity and selectivity. A prerequisite for such research is synthesis methods that enable fabricating catalysts with control over the exposed active sites. Area-selective ALD [33], whereby a material is only deposited on a selected area of a substrate, is ideal for that purpose. By only covering specific facets, edges, or corners of a catalyst particle with a functional element, it enables directional and precise tailoring of the structural parameters, interfaces, and thus also the active sites.

The deposition selectivity during such ALD processes originates from the quick material growth on a specific site or surface, while there is a nucleation delay before growth initiation on other, less desired sites or surfaces [33]. In inherently selective ALD, this is established by an appropriate combination of precursor, co-reactant, substrate material, precursor partial pressure and deposition temperature [307]. Alternatively, the deposition can be preceded by a surface preparation treatment prior to the ALD process to selectively functionalize the sample. Examples thereof include the use of long-chain molecules [308] or plasmas [309] to passivate or activate particular surfaces of the NP in order to specifically avoid or target deposition on said surfaces. Moreover, the aforementioned functionalization step can be repeated, either separately as a wet chemical step after a set number of ALD cycles [310] or by incorporating it as a vapor phase step into each ALD cycle [311]. Another approach to improve area-selectivity includes selective etching after ALD to remove deposited material from specific areas [312]. However, it is also possible to implement this etching into the ALD process. For instance, this can be implemented through repeated exposure to ALD and etching cycles or so-called “supercycles” [313,314].

Over the past decade, ALD protocols for the accurate deposition of various metals and oxides have already set course for next-level catalyst design [37,38,39]. Some of the earliest examples include the creation of core-shell structures by limiting the deposition of metals (or metal oxides) strictly to NPs instead of the entire surface [221,222,252,308]. Area-selective ALD has also been successfully applied to fabricate novel 3D nanostructures, such as “nanotraps” [315], whereby a NP is ‘trapped’ in a ‘pit’ of oxide material. This is implemented by targeting deposition on the support surrounding the NP. One step beyond area-selective ALD is the deposition of a functional element onto specific planes/facets/edges of a NP, i.e., site-selectivity as the most recent ALD application [316,317,318,319]. Depending on the coating material, different functionalities can be targeted, such as: passivation or site blocking [318], which is of major interest for site-specific studies; activation [319]; localized alloy formation; or carbon control.

The growing interest in site-selective ALD is demonstrated by recent theoretical-experimental work. Cao et al. [320] researched facet-selective ALD using thd precursors (thd = 2,2,6,6-tetramethylheptane-3,5-dione), where CeO_x_ was selectively deposited on (111) facets of Pt NPs to leave the Pt(100) facets naturally exposed. The facet-selectivity is implemented through different binding energies of the thd-based precursor fragments, chemisorbed onto Pt(111) or Pt(100), resulting in a ceria “nanofence” structure with ceria coated Pt(111) facets, where the ceria-metal interfaces can enhance activity and delay sintering, while the Pt(100) facets remain exposed and easily accessible for e.g., CO oxidation. More recently, Wen and co-workers [321] combined DFT calculations with microkinetic methods to investigate the edge-selective ALD growth mechanism of 3d-transition metal oxides on Pt NPs in ALD based on metal cyclopentadienyl (Cp) precursors. MCp_2_ (where M = Fe, Ni or Co) decomposition on the Pt NPs’ surface exhibits strong preferential growth, following the order of edge > (100) > (111). The preferred deposition on the edge site is attributed to a more favorable precursor splitting pathway.

**Figure 21:**
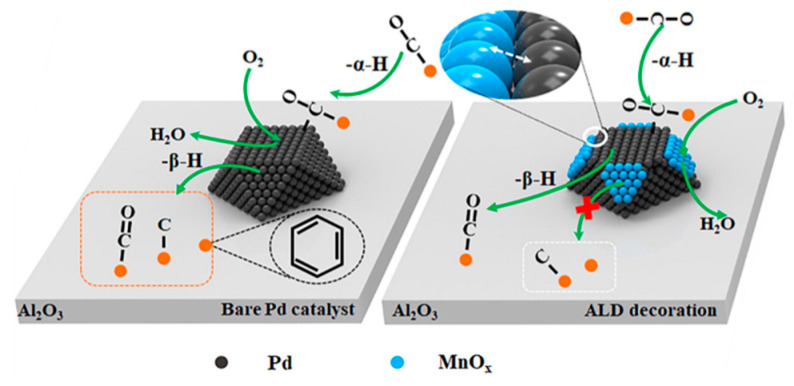
Schematic representation of the structure-property mechanism of (MnO_x_/)Pd/Al_2_O_3_ for benzaldehyde oxidation. For unmodified Pd/Al_2_O_3_ (left) benzyl alcohol is first adsorbed on Pd^0^, and a Pd atom is located between the O-H of the alcohol to form a metal alkoxide and a metal hydride (adsorbed on adjacent Pd atoms); then, a hydrogen atom is removed by the surface Pd atom through the β-H elimination process to form benzaldehyde; finally, the adsorbed hydrogen is oxidized by absorbed oxygen to regenerate the Pd active sites. For ALD MnO_x_-modified Pd/Al_2_O_3_ (right) molecular oxygen is adsorbed on MnO_x_ and activated to adsorb the H atom of the metal alkoxide formation process and the H atom of the β-H elimination process to form benzaldehyde and H_2_O. Selective Pd(111) facet passivation eliminates the formation of toluene via decarbonylation of benzaldehyde. Concomitantly, benzene formation over these facets is decreased. Reprinted with permission from Ref. [323]. Copyright 2020 Elsevier.

Dedicated reactivity studies have been reported on site-selective ALD-prepared materials. Though initial studies were limited to planar substrates [318,320]—due to their compatibility with ALD processes and surface characterization techniques—applications are already advancing to high surface area supports [316,317,319]. Wang et al. [322] utilized Al_2_O_3_ and FeO_x_ ALD to selectively block high- and low-coordination sites on the Pd NPs of Pd/Al_2_O_3_. Complemented with DFT calculations, their approach allowed to disentangle the geometric and electronic effects of the NPs on the catalytic performance in solvent-free oxidation of benzyl alcohol. In recent progress, Hu et al. [323] applied MnO_x_ ALD on WI-prepared Pd/Al_2_O_3_ to selectively passivate Pd(111) facets. This prevented the formation of toluene by-product by eliminating decarbonylation of benzaldehyde, which would otherwise occur over these facets (Figure 21). In addition, the facet passivation decreased benzene formation. Moreover, relative to uncovered Pd/Al_2_O_3_, the MnO_x_ species introduced O migration at MnO_x_–Pd interfaces and increased the concentrations of Pd^0^ sites, which are the active sites for benzyl alcohol adsorption. Altogether, these factors improved the TOF value with a factor 8.7 and increased the benzyl alcohol conversion and benzaldehyde yield with 84.7% and 76.5%, respectively.

It can be concluded that area-selective ALD is an emerging topic within catalyst synthesis. Specifically, site-selective ALD holds enormous potential for the atomic-scale design and systematic structure-activity study of catalysts. Though the site-selective ALD technique is still in its infancy, as indicated by the fundamental studies on this topic, the opportunities it offers for the nanoengineering of catalysts make it promising to bring the understanding of catalysis to the next level.

## 4. Conclusions

The creation of well-defined NPs supported on porous supports is fundamental for the rational design of catalysts. In that regard, bottom-up colloidal synthesis techniques as well as ALD have been successful in creating such uniform materials in view of their application in the gas-phase catalysis of various reaction families. As demonstrated in this review, studies have focused on supported monometallic, bimetallic NPs, as well as particulates embedded into the support or coated with porous oxide overlayers. Herein, both synthesis techniques have proven their worth in fundamental studies whereby the role of structure, composition, and the nature of the active site have been elucidated.

While colloidal techniques and ALD provide opportunities for controlled catalyst design, advantages and disadvantages can be distinguished for each. These are summarized in Table 1. When economics are not accounted for, decisive factors in choosing one of either techniques include the complexity of equipment-related requirements as well as the ease of application in the generation of NPs of a certain (or multiple) element(s).

## 5. Outlook: Colloidal Synthesis and ALD Applied to Heterogeneous Catalysis

The controlled synthesis of supported NPs and their catalytic applications is an ever-growing discipline, such that future improvements are expected regarding the synthesis of novel materials, their characterization and the technologies involved in their fabrication.

Not taking the support or possible oxidic overlayers into account, controlled materials for gas-phase catalysis are to-date limited to mono- and bimetallic materials. While some tri- and multimetallic supported materials have been prepared using colloids [47] and ALD [324], associated protocols are not as abundant as for mono- and bimetallic materials. In addition, most studies are focused on the synthesis of these materials rather than on applying them. In the select application studies that exist, gas-phase catalysis has not been researched [47,324,325,326,327]. More so, in the case of ALD, these materials are limited to non-porous substrates [324]. Hence, future studies may consist in (1) the synthesis of novel tri- and multimetallic supported catalysts via colloidal synthesis and (2) ALD, preferably on porous supports, as well as (3) their catalytic applications. Although various mono- and bimetallic systems have been explored through the use of controlled synthesis techniques, there are still many industrially relevant mono- and bimetallic catalysts which have only been examined via conventional and uncontrolled synthesis techniques. As a result, these latter catalysts can profit from the use of colloidal synthesis or ALD in extending the fundamental understanding of their properties and aid in the rational design of desirable catalytic sites. Of course, this necessitates the further development of colloidal and ALD protocols that can effectively create the above-mentioned materials. With the goal of cost minimization, related future work is best focused on non-noble metals.

In the fabrication of the abovementioned materials, general directions can be given for advanced synthesis strategies, that allow improved control over the nanoparticulate materials. One such strategy includes the exploitation of MSI. It has been indicated throughout this review that MSI play a pivotal role in determining the catalytic properties both during synthesis and reaction, e.g., by mitigating NP migration through strong interaction between support and metal. Nevertheless, systematic knowledge of the factors influencing MSI is lacking to-date, which instigates dedicated studies to investigate these factors in order to allow tailoring MSI for improved control of the catalytic performance. The use of embedded or coated NPs presents another opportunity. While such materials have proved effective in improving catalytic stability relative to bare NPs, the coating layers often limit access to the surface of the metals contained within. Hence, future work should focus on overcoming this shortcoming, for example, through the development of colloidal or ALD synthesis protocols which do not introduce this bottleneck. For colloidal approaches, this requires the development of synthesis methods to further enhance the control over the spatial distribution, architecture and porosity of the oxide phase surrounding the active NPs. In relation to ALD, MLD-based approaches where conformal deposition of a hybrid coating followed by calcination can result in a conformal, microporous oxide shell, offers promise for achieving ultra-thin, conformal coatings, with atomic level thickness control as well as offering tailored microporosity. Particularly interesting for catalytic applications, though not yet implemented, is the use of metalcone MLD whereby the use of larger organic template molecules can be used to provide sufficient access to the metal sites of the catalyst.

Within ALD, the concept of area-selective ALD has potential for the atomic-scale fabrication of catalysts. Specifically, site-selective ALD to either block or activate/promote specific catalyst sites holds great promise to target enhanced activity and selectivity in future (site-specific) catalytic studies. The select, yet recent, number of studies on this subject are proof of the growing interest for this branch of ALD. Related future work may therefore consist in the development of novel selective ALD procedures, as well as their applications in catalysis.

As illustrated in this review, the use of established ex situ, in situ and operando techniques in catalytic studies with controlled NPs has already proved its value in attaining unrivaled fundamental understanding of the factors that influence catalytic performance. To bring this understanding via these fine-tuned materials to the next level, studies must move more towards in situ and operando techniques to allow unbiased tracking of geometrical and electronic changes that take place during the reaction and correlate these with the catalytic performance. However, as the active sites are only present in small quantities and their signature is often dominated by larger spectator contributions, it remains a challenge for in situ and operando characterization to investigate the real catalyst species with high selectivity. In that regard, the application of stimulated changes in the reactive atmosphere via modulation-excitation (ME) [328] has received strong attention to selectively identify the active sites on supported NPs. While ME has been successfully applied to XAS [329,330], as well as infrared spectroscopy [331], Raman spectroscopy [332] and XRD [333], it has to-date been little employed to study controlled materials prepared by colloidal synthesis or ALD. Hence, there is much untapped potential for well-defined systems through such in situ/operando experiments. Moreover, in situ kinetic studies that are (partially) transient in nature, such as temporal analysis of products (TAP) [334] or steady-state isotope transient kinetic analysis (SSITKA) [335], are little to not reported for well-defined materials [336]. Nevertheless, applying these techniques to controlled materials can give additional fundamental catalytic insights, especially when combined with the above-mentioned characterization techniques.

While catalysts fabricated via colloidal approaches or ALD are promising, related synthesis procedures are currently limited to lab-scale batch processes. Large-scale colloidal synthesis is hampered by the high cost of commonly used organometallic precursors, the large solvent volumes used to obtain high particle dilution and the lack of scalable NP stabilization methods [21]. In the case of ALD, the equipment and precursor costs are the limiting factors. However, as ALD is commercially applied in the microelectronics industry and reactors for large-scale deposition [212] and continuous operation [337,338] have been proposed, its future industrial scalability can be deemed more feasible. Still, the significant cost of equipment and precursor is only justified if the end-product, i.e., the catalyst, has sufficient added value. The high initial activity and stability of materials prepared via ALD already contribute to its high value. Moreover, the addition of only a few cycles of oxide coating can improve the stability, which reduces the material cost. Together with its atomic-level degree of control, these ‘high-value factors’ could facilitate large-scale controlled catalyst synthesis by ALD.

Even if commercial scale-up of controlled catalyst synthesis is never attained for either colloidal synthesis or ALD, both methods have, as demonstrated in this review, already proved their worth in research for a wide variety of fundamental studies. It is thus expected that both methods shall continue to be employed for future studies, not only for academic purposes, but also to examine improvements for potential future industrial applications.

## Figures and Tables

**Table 1:** Summarized advantages (+) and disadvantages (−) of colloidal synthesis and ALD for supported catalyst synthesis.

	Colloidal Synthesis		ALD	
	+	−	+	−
**Equipment**	Simple equipment (hot plate, stirrer, inert gas/vacuum line)			Requires dedicated setups/reactors ^1^; (vacuum equipment) ^2^; deposition onto porous supports requires dedicated equipment to assure adequate exposure of all surfaces
**Chemicals:** **-precursor**	Broad choice of precursor type			Requires volatile precursors^1^
**-solvent**		Requires solvent (removal)	No solvent (removal) required ^2^	
**-stabilizing** **agent**		Removal of stabilizing agent required by thermal process	No stabilizing agent removal required; thermal activation processes not per se necessary ^3^	
**Control of:** **-size**	NP size easily tunable down to ~nm size		Å-level control of coatings/NP size	
**-composition**	Can prepare bi/tri/multimetallic NPs of virtually any elements, provided precursors exist			Creation of bi/tri/multimetallic NPs requires precursors with compatible reaction chemistry
**-coating**		Embedding restricted to colloids	Oxide overlayer deposition possible	
**Deposition:** **-on support**		Requires a NP deposition step ^4^	Direct deposition on substrate	
**-selective**		Not selective	Chemical selectivity (area-selective ALD)	
**-conformal**		Deposition into high surface area materials depends on several factors: e.g., synthesis method, pore size, NP size	Deposition possible into mesoporous materials as long as the pore diameter is larger than the size of the precursor molecule	

^1^ These factors introduce significant costs relative to colloidal procedures. ^2^ Although recent developments in ALD from solvents have been reported, ALD under vacuum has been the prevalent type used for catalyst synthesis. ^3^ Exceptions include calcination treatments after ALD of oxide overlayers to introduce porosity, and ALD processes where the deposited phase does not correspond to the active phase for reaction (e.g., a H_2_ treatment after metal oxide ALD). ^4^ Exceptions include in situ reduction methods.

## Acronyms

**3DOM**	three-dimensionally ordered macroporous
**ALD**	atomic layer deposition
**BDE**	1,3-butadiene
**BTB**	borane tert-butylamine complex
**CNT**	carbon nanotube
**Cp**	cyclopentadienyl
**CVD**	chemical vapor deposition
**DFT**	density functional theory
**DME**	dimethyl ether
**DP**	deposition-precipitation
**DRM**	dry reforming of methane
**FTO**	Fischer Tropsch to olefins
**FTS**	Fischer Tropsch synthesis
**GC**	graphitic carbon
**GHSV**	gas hourly space velocity
**GISAXS**	grazing incidence small-angle X-ray scattering
**H_2_-TPR**	hydrogen temperature-programmed reduction
**HAADF**	high-angle annular dark-field
**H-Al_2_O_3_**	hydrophobic Al_2_O_3_
**h-BN**	hexagonal boron nitride
**HDP**	homogeneous deposition-precipitation
**IR**	infrared
**IWI**	incipient wetness impregnation
**ME**	modulation-excitation
**MeOH**	methanol
**MLD**	molecular layer deposition
**MOF**	metal-organic framework
**MSI**	metal-support interactions
**m-SiO_2_**	mesoporous SiO_2_
**NAP-XPS**	near-ambient pressure X-ray photoelectron spectroscopy
**NEXAFS-TEY**	near edge X-ray absorption fine structure spectroscopy with total electron yield
**NMR**	nuclear magnetic resonance
**NP**	nanoparticle
**OAm**	oleylamine
**P**	pressure
**PAMAM**	polyamidoamine
**PDF**	pair distribution function
**PDH**	propane dehydrogenation
**PODH**	propane oxidative dehydrogenation
**PROX**	preferential oxidation of CO in H_2_
**PVA**	polyvinyl alcohol
**RCT**	raspberry colloid-template
**SRM**	steam reforming of methane
**SSITKA**	steady-state isotope transient kinetic analysis
**STD**	syngas-to-dimethyl
**STEM-EDX**	scanning transmission electron microscopy energy dispersive X-ray
**T**	temperature
**TAP**	temporal analysis of products
**TEM**	transmission electron microscopy
**TGA**	thermogravimetric analysis
**thd**	2,2,6,6-tetramethylheptane-3,5-dione
**TMA**	trimethylaluminum
**TOF**	turnover frequency
**TOS**	time-on-stream
**TTAB**	trimethyl(tetradecyl)ammonium bromide
**UHV**	ultra-high vacuum
**V**	volume
**WHSV**	weight hourly space velocity
**WI**	wet impregnation
**XAS**	X-ray absorption spectroscopy
**XPS**	X-ray photoelectron spectroscopy
**XRD**	X-ray diffraction

## Glossary

**activation (catalyst)**	The procedure whereby a catalyst is put into a state (activated state) which can catalyze the reaction. Depending on the reaction at hand, this can be, for example, reduction (active phase = metal) or sulfidation (active phase = sulfide).
**active site**	An ensemble of atoms on the catalyst which directly catalyzes a reaction.
**activity (catalyst)**	Catalytic activity refers to the reaction rate, i.e., the speed at which a reaction takes place. This is typically quantified by turnover frequency (TOF).
**calcination**	The process of heating a sample, in this context a catalyst, to high temperature under an oxygen or air environment.
**cavitation bubble**	A small bubble, typically with sizes on the order of µm–mm, that can collapse explosively and generate a localized increase in pressure (e.g., shock waves) and temperature. In the case of sonication (ultrasound) of a liquid, these bubbles originate from rapid compressions and expansions of the liquid medium.
**chemical reduction (colloidal synthesis)**	A colloidal synthesis protocol whereby reduction to zerovalent state of the metal is achieved-in part-through the addition of a reducing agent.
**coking**	The process whereby cokes are formed, i.e., carbonaceous species, as undesired by-products of a reaction. As cokes occupy active sites, this leads to catalyst poisoning.
**colloid**	A type of mixture whereby solid or liquid particles, in the range of 1–1000 nm, are dispersed in a liquid phase and surrounded by a protective layer which prevents their agglomeration.
**conformal**	With even thickness over the exposed surface.
**conversion (of a reagent)**	In reaction engineering, the conversion of a reagent is defined as the ratio of [the number of moles of a certain reagent that have reacted] over [the initial number of moles of reagent].
**core-shell**	An architecture consisting of a core material (A) surrounded by a concentric shell of a second material (B). Typically denoted as A@B.
**crystal facet**	A planar side of a geometrical shape.
**deactivation (catalyst)**	The process whereby a catalyst loses its activity over time. Typical deactivation phenomena include coking and metal sintering.
**embedded nanoparticles**	Nanoparticles which are located within the support material, rather than on the support’s surface.
**in situ reduction (colloidal synthesis)**	Colloidal deposition protocol whereby the colloidal synthesis takes place in the presence of the support.
**incipient wetness impregnation**	Catalyst synthesis protocol whereby the metal precursor is first dissolved in a solvent, after which this dissolved precursor is added to the support. Herein, the volume of solution used is the same as the support pore volume.
**Janus-like architecture**	Nanoparticle architectural pattern that exhibits two distinguishable subunits. For instance, in a bimetallic CoFe particle, one subunit can exhibit low Co concentration, whereas the other subunit exhibits much higher Co concentration.
**mesoporous**	Containing pores with pore diameters between 2 and 50 nm.
**metal precursor**	A compound that contains a metal in its ionic state and serves as the metal source for catalyst synthesis.
**microporous**	Containing pores with pore diameters smaller than 2 nm.
**monodisperse distribution**	Displaying a narrow distribution. Synonym for uniform.
**multimetallic**	In this paper, multimetallic refers to any composition that contains more than 3 metals.
**nanoparticle**	A particle in the nanoscale regime, i.e., 1–100 nm.
**nucleation**	The initial process whereby a novel phase starts growing within or onto a pre-existing phase.
**organometallic salt**	A substance which is partially ionic (in the case of a metal precursor, this is a metal ion) and partially organic. An example is Ni(acac)_2_, made up of Ni^2+^ with surrounding (organic) acetylacetonate (acac) groups.
**performance (catalyst)**	A catalyst’s performance refers to its behavior during reaction. Commonly quantified via the terms reagent conversion, product selectivity, product yield, activity and stability.
**poisoning (catalyst)**	The act of blocking active sites of a catalyst with unwanted material, e.g., cokes, thus inhibiting contact of these active centers with reagents, which is detrimental to the catalytic activity.
**promoter (catalyst)**	A substance added to a catalyst to improve its performance.
**protective agent**	A chemical agent used in colloidal synthesis. It has a dual purpose: controlling the nanoparticle growth (capping agent) and preventing coalescence of colloidal nanoparticles (stabilizing agent).
**rational/knowledge-driven catalyst design**	The design of novel and improved catalysts in an efficient and cost-effective way. The creation of monodisperse materials helps to achieve this goal as follows. Materials with well-defined properties (e.g., nanoparticle size) allow investigating the effect of this property on the catalytic performance for a given reaction. As such, the optimal catalyst property (e.g., size which gives maximum activity) can be identified, which gives guidelines (knowledge) for the design of a new and improved catalyst.
**reactant (ALD)**	One of the two main components, besides a metal precursor, making up an ALD cycle. Reactants typically are gases, e.g., H_2_O, NH_3_, H_2_S, O_3_ etc., which, unlike the metal precursor, do not contain any metals.
**reducing agent**	A chemical added during colloidal synthesis (in chemical reduction protocols) which helps implement the reduction of the metal ions to their zerovalent state
**selectivity (towards a product)**	In reaction engineering, the conversion of a reagent is defined as the ratio of [the number of moles of the product that have formed] per [mole of reagent that has reacted].
**self-limiting surface reaction**	A surface reaction which stops when the surface is depleted of functional groups.
**single crystal**	A solid wherein an orderly three-dimensional arrangement of atoms is repeated throughout the entire material.
**sintering**	The thermally-induced process whereby small particles coalesce to form bigger particles. When this occurs for the nanoparticles in a catalyst, this leads to lower surface area of the active phase.
**stability (catalyst)**	A term denoting to what degree a catalyst retains its (initial) performance after a certain reaction time. Typically measured by monitoring the performance over an extended reaction time and observing the changes in activity/selectivity/conversion/etc. relative to initial values.
**structure-sensitive reaction**	A reaction of which the reaction kinetics are dependent on the surface structure of the catalyst.
**support**	A solid material, onto which the active nanoparticles are fixed, supplying mechanical strength and a large area to disperse the active phase. Sometimes also called substrate, though this latter term is more used in the context of planar support materials.
**thermal decomposition (of an organometallic metal precursor)**	A type of colloidal synthesis protocol whereby the metal precursor is organometallic in nature, e.g., an organometallic salt, and, through exposure to high temperatures, is decomposed and (thermally) reduced to its zerovalent state.
**turnover frequency**	A measure used to quantify the number of reagent conversions an active site performs per second.
**wet impregnation**	Catalyst synthesis protocol whereby the metal precursor is first dissolved in a solvent, after which this dissolved precursor is added to the support. Herein, the volume of solution used is much larger than the support pore volume.
**yield (of a product)**	In reaction engineering, the yield of a product is defined as the ratio of [the number of moles of the product that have formed] over [the initial number of moles of reagent]. Mathematically, this follows from multiplying the reagent conversion with the product selectivity.
